# Clinical Correlations of Polycomb Repressive Complex 2 in Different Tumor Types

**DOI:** 10.3390/cancers13133155

**Published:** 2021-06-24

**Authors:** Maksim Erokhin, Olga Chetverina, Balázs Győrffy, Victor V. Tatarskiy, Vladic Mogila, Alexander A. Shtil, Igor B. Roninson, Jerome Moreaux, Pavel Georgiev, Giacomo Cavalli, Darya Chetverina

**Affiliations:** 1 Group of Chromatin Biology, Institute of Gene Biology, Russian Academy of Sciences, 34/5 Vavilov Street, 119334 Moscow, Russia; yermaxbio@yandex.ru (M.E.); chetverina.oa@phystech.edu (O.C.); 2 Moscow Institute of Physics and Technology, 9 Institutskiy Pereulok, Dolgoprudny, 141701 Moscow, Russia; 3 Institute of Electronic Control Machines Named after I. S. Brook (INEUM), 24 Vavilov Street, 119334 Moscow, Russia; 4 TTK Cancer Biomarker Research Group, Institute of Enzymology, Magyar Tudosok Korutja 2, 1117 Budapest, Hungary; gyorffy.balazs@ttk.hu; 5 Department of Bioinformatics and 2nd Department of Pediatrics, Semmelweis University, Tuzolto u. 7–9, 1094 Budapest, Hungary; 6 Laboratory of Molecular Oncobiology, Institute of Gene Biology, Russian Academy of Sciences, 34/5 Vavilov Street, 119334 Moscow, Russia; Tatarskii@genebiology.ru (V.V.T.); shtil@scamt-itmo.ru (A.A.S.); 7 Department of Control of Genetic Processes, Institute of Gene Biology, Russian Academy of Sciences, 34/5 Vavilov Street, 119334 Moscow, Russia; v@mogila.org (V.M.); georgiev_p@mail.ru (P.G.); 8 Department of Drug Discovery and Biomedical Sciences, University of South Carolina, 715 Sumter Street, Columbia, SC 29208, USA; roninsoni@cop.sc.edu; 9 Institute of Human Genetics, UMR 9002 Centre National de la Recherche Scientifique, University of Montpellier, 34396 Montpellier, France; jerome.moreaux@igh.cnrs.fr; 10 Department of Biological Hematology, CHU Montpellier, 34295 Montpellier, France; 11 UFR Medicine, University of Montpellier, 34003 Montpellier, France; 12 Institut Universitaire de France (IUF), 75005 Paris, France; 13 Group of Epigenetics, Institute of Gene Biology, Russian Academy of Sciences, 34/5 Vavilov Street, 119334 Moscow, Russia

**Keywords:** PRC2, EZH2, cancer, oncology, PRC2 inhibitors, EZH2 inhibitors, biomarkers, SWI/SNF, SMARCB1, BCL2, Polycomb

## Abstract

**Simple Summary:**

PRC2 (Polycomb repressive complex 2) is a catalytic multi-subunit complex involved in transcriptional repression through the methylation of lysine 27 at histone 3 (H3K27me1/2/3). Dysregulation of PRC2 has been linked to tumor development and progression. Here, we performed a comprehensive analysis of data in the genomic and transcriptomic (cBioPortal, KMplot) database portals of clinical tumor samples and evaluated clinical correlations of EZH2, SUZ12, and EED. Next, we developed an original Python application enabling the identification of genes cooperating with PRC2 in oncogenic processes for the analysis of the DepMap CRISPR knockout database. Our study identified cancer types that are most likely to be responsive to PRC2 inhibitors. By analyzing co-dependencies with other genes, this analysis also provides indications of prognostic biomarkers and new therapeutic regimens.

**Abstract:**

PRC2 (Polycomb repressive complex 2) is an evolutionarily conserved protein complex required to maintain transcriptional repression. The core PRC2 complex includes EZH2, SUZ12, and EED proteins and methylates histone H3K27. PRC2 is known to contribute to carcinogenesis and several small molecule inhibitors targeting PRC2 have been developed. The present study aimed to identify the cancer types in which PRC2 targeting drugs could be beneficial. We queried genomic and transcriptomic (cBioPortal, KMplot) database portals of clinical tumor samples to evaluate clinical correlations of PRC2 subunit genes. *EZH2*, *SUZ12*, and *EED* gene amplification was most frequently found in prostate cancer, whereas lymphoid malignancies (DLBCL) frequently showed *EZH2* mutations. In both cases, PRC2 alterations were associated with poor prognosis. Moreover, higher expression of PRC2 subunits was correlated with poor survival in renal and liver cancers as well as gliomas. Finally, we generated a Python application to analyze the correlation of *EZH2/SUZ12/EED* gene knockouts by CRISPR with the alterations detected in the cancer cell lines using DepMap data. As a result, we were able to identify mutations that correlated significantly with tumor cell sensitivity to PRC2 knockout, including SWI/SNF, COMPASS/COMPASS-like subunits and BCL2, warranting the investigation of these genes as potential markers of sensitivity to PRC2-targeting drugs.

## 1. Introduction

The maintenance of tissue-specific gene expression profiles is required for a normal development and physiology of multicellular organisms. In particular, epigenetic control of transcriptional repression is implemented by the Polycomb group (PcG) proteins that function as a part of multiprotein complexes recruited to the chromatin [1,2,3,4,5]. The Polycomb repressive complex 2 (PRC2) in mammals consists of three core subunits: EZH2 (Enhancer of Zeste Homolog 2), SUZ12 (Suppressor of Zeste 12), and EED (Embryonic Ectoderm Development) proteins [6,7,8]. The enzymatic component of the complex is EZH2, a histone methyltransferase (HMT) that catalyzes an addition of up to three methyl groups to the histone H3 lysine 27 (H3K27me1/2/3) via its SET domain [9,10,11,12]. The HMTase activity of EZH2 requires the presence of the SUZ12 and EED subunits [13,14,15].

The PRC2 complex is vital for mammalian development. Mouse embryos with deletions in the *EZH2*, *EED* or *SUZ12* genes fail to develop and die during the post-implantation period [15,16,17], while *EZH2*^−/−^ human ESCs (hESCs) demonstrate self-renewal and differentiation defects [18].

Numerous studies have shown that PRC2 is broadly implicated in cancer biology, and alterations of its subunits can be associated with poor survival [2,4,19,20,21,22,23]. These alterations include both the overexpression of PRC2 genes and mutations that either enhance or inhibit PRC2 catalytic activity. EZH2 overexpression is frequently accompanied by amplification of the *EZH2* gene; less is known about SUZ12 and EED. The suppression of PRC2 activity has been shown to inhibit the growth of certain tumors. Several small molecule inhibitors targeting PRC2 have been developed; some are undergoing clinical trials [24,25]. The first-in-class compound tazemetostat targeting the EZH2 protein was recently approved in the USA for the treatment of patients with metastatic or locally advanced epithelioid sarcoma [26,27,28] and for adult patients with relapsed or refractory (R/R) follicular lymphoma (FL) [29]. In epithelioid sarcoma, the sensitivity to tazemetostat is associated with a loss of SMARCB1, a component of the SWI/SNF remodeling complex of Trithorax group (TrxG) proteins that antagonize PcG repression [27].

Despite the progress made in recent years, many aspects of the role of PRC2 in cancer remain unaddressed. Are SUZ12 and EED implicated in various tumor types? What are the clinical implications of different PRC2 subunits? What other mutations, besides for the SWI/SNF members, could confer tumor sensitivity to inhibitors of PRC2 subunits and could be used as clinically relevant markers?

In the present study we analyzed publicly available genomic and transcriptomic database portals to identify cancers in which the PRC2 subunits are likely to play essential roles.

## 2. Materials and Methods

The cBioPortal for cancer genomics (https://www.cbioportal.org/, accessed on 2 November 2020) [30] was used to query alterations in *EZH2/SUZ12/EED* genes in clinical samples. DNA analysis was carried out using a set of 185 studies (TCGA- and non-TCGA) that were manually curated with no overlapping samples (total 48,045 samples). In the analysis of the frequency of a specific alteration, only samples tested with available information (e.g., the copy number or missense/gain-of-function (GOF) mutations) were taken into account.

For multiple myeloma survival analysis, we used RNAseq data of 674 newly diagnosed MM patients with longitudinal follow-up from the Multiple Myeloma Research Foundation CoMMpass trial (NCT01454297; version IA11a), termed in the following CoMMpass cohort [31]. The statistical significance of differences in overall survival between patients’ groups was calculated using the log-rank test and survival curves were plotted using the Kaplan-Meier method. For other tumor types, the survival analysis was carried out using the Pan-Cancer datasets of the online tool www.kmplot.com (accessed on 15 January 2021) [32]. The Pan-Cancer dataset is based on TCGA data generated using the Illumina HiSeq 2000 platform with survival information derived from the published sources [33]. In the survival analysis, each cutoff between the lower and upper quartiles was analyzed by Cox proportional hazards regression; the best performing cutoff was used in the final analysis. The Kaplan-Meier survival plots were generated, and the hazard rates with 95% confidence intervals were computed to numerically assess the difference between the two cohorts.

DepMap (https://depmap.org/portal/, accessed on 6 October 2020) analysis of the dependency of a panel of tumor cell lines on individual genes was conducted using the CRISPR (Avana) Public 20Q3 (DepMap, Broad (2020): DepMap 20Q3 Public. figshare. Dataset doi:10.6084/m9.figshare.12931238.v1.) [34,35]. The following files of the Public 20Q3 release were downloaded for the analysis: “Achilles_gene_effect.csv” (Genetic Dependency CRISPR (Avana) Public 20Q3, Genes 18119, 789 Cell lines, 30 Primary Diseases, 27 lineages); ”CCLE_mutations.csv” (Cellular Models Mutation Public 20Q3, 18802 Genes, 1741 Cell Lines, 35 Primary Diseases, 37 Lineages); “sample_info.csv” (Cellular Models Cell Line Sample Info, 1804 Cell lines, 35 primary diseases, 38 lineages). For gene ontology data the Uniprot (https://www.uniprot.org/, accessed on 22 February 2021) database was used. The Power Query Microsoft Excel Tool was used to combine the data from different files. In total, the DepMap 20Q3 release contains the results of the experimental analyses of the effects of *EZH2*, *SUZ12* or *EED* gene knockout by the CRISPR/Cas9 method on the proliferation of 777 cancer cell lines (Supplementary File S1 “CRISPR (AVANA) Public 20Q3 PRC2 cell lines info”).

To calculate *p*-values for the dependence hypotheses between the sensitivity of cell lines to *EZH2*, *SUZ12,* or *EED* knockouts and the presence of each documented non-silent mutation, we developed an original application termed Genes.py. Genes.py is written in Python and uses the Scipy, Numpy, and Pandas libraries. The silent mutations were excluded from the analysis using the attribute ‘Variant_Classification’ in ‘Cellular Models Mutation Public 20Q3′ file. Source code of the analysis is available at the following link: https://github.com/genesolution/PRC2_data (uploaded on 25 March 2021).

## 3. Results

### 3.1. Analysis of EZH2, SUZ12, and EED DNA Alterations in Patients’ Samples

Depending on the cancer type, alterations in the PRC2 methyltransferase complex have been demonstrated to be both pro- and anti-oncogenic [36]. Candidate cancers that might be sensitive to PRC2 inhibitors are those in which PRC2 plays an oncogenic function. These are the tumors characterized by overexpression of PRC2 encoding genes or by GOF mutations in the *EZH2* gene leading to an increased PRC2 catalytic activity [2,19,20]. Amplification of the *EZH2* coding region has been detected in different tumors [37,38,39,40]. Similarly, the *SUZ12* gene is amplified in several tumors [41], nevertheless, there is a lack of information regarding the *SUZ12* and *EED* amplification. Furthermore, amplification frequencies of individual PRC2 components have not been described.

**Figure 1:**
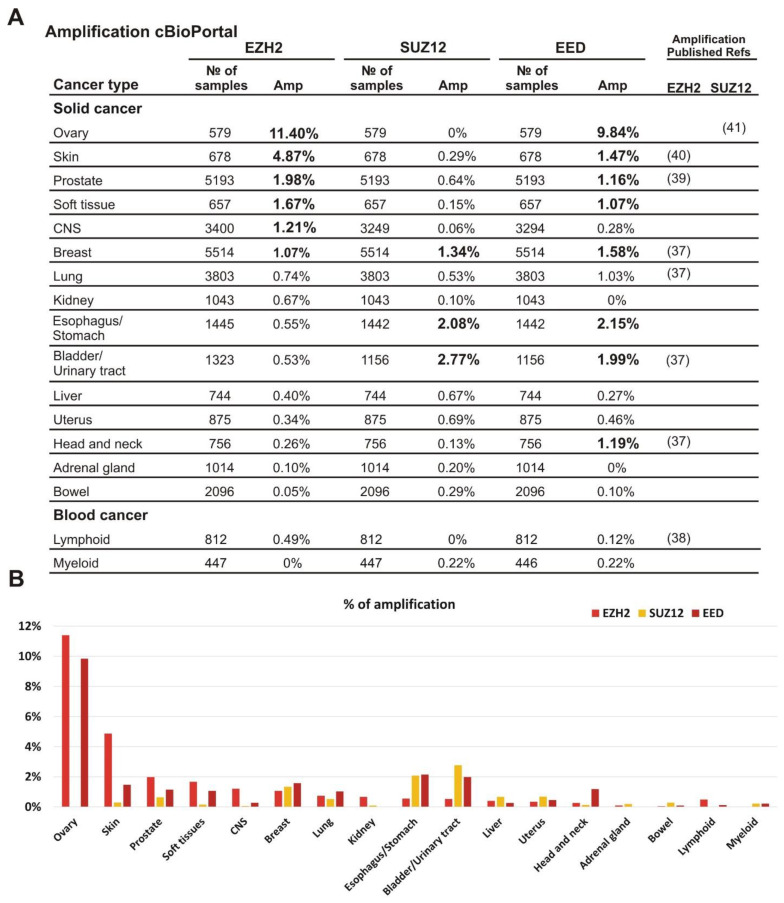
Amplification of *EZH2*, *SUZ12* and *EED* is frequently present in clinical cancer samples. (**A**) The analysis was performed using the cBioPortal database, which includes 185 studies and 48,045 non-overlapping samples. The cancer types containing more than 500 samples in the database are listed. The exact number of samples analyzed and the percent of cases with amplification is shown for each cancer type. All values above 1% are shown in bold; for convenience. The references to previous studies identifying amplification for *EZH2* or *SUZ12* are indicated on the left. (**B**) Graphical representation of *EZH2*, *SUZ12* and *EED* frequencies of the amplifications.

To characterize the amplification of *EZH2*, *EED*, and *SUZ12* in different tumor types we analyzed clinical samples for the amplified PRC2 subunit genes using the cBioPortal database (https://www.cbioportal.org/, accessed on 2 November 2020) [30]. The samples were grouped by tissue origin and only cohorts with at least 500 samples were analyzed. We found that the amplification of *EZH2* as well as *EED* and *SUZ12* subunits was not limited to a specific tumor type (Figure 1A,B). Furthermore, the amplification frequency of specific subunits differed significantly among tumor types (Figure 1A,B). Interestingly, simultaneous amplification of several PRC2 subunit genes can be detected in the same clinical sample (Supplementary File S2).

The most frequent *EZH2* amplification (11.4%) was observed in ovarian cancer. In this tumor type, *EED* was amplified in 9.84% of cases, while amplification of the *SUZ12* was not observed. Similar tendency for the higher rate of *EZH2* and *EED* amplification was found for skin, prostate, and soft tissue tumors (Figure 1A,B). Breast carcinomas showed approximately similar amplification rates for each of the three PRC2 genes. In CNS tumors, a higher amplification was observed for *EZH2*, while bladder/urinary tract and esophagus/stomach cancers displayed a more frequent amplification of *EED* and *SUZ12*. Head and neck tumors demonstrated a higher level of *EED* amplification than *EZH2* and *SUZ12*. The adrenal gland and bowel cancers, as well as lymphoid and myeloid malignancies showed the lowest percentage of cases with amplification (Figure 1A,B). Thus, although the amplification of the *EZH2, EED,* and *SUZ12* genes encoding the PRC2 subunits was detectable in the tumors derived from different tissues, the combinations of amplified genes and the frequency of this alteration varied significantly.

**Figure 2:**
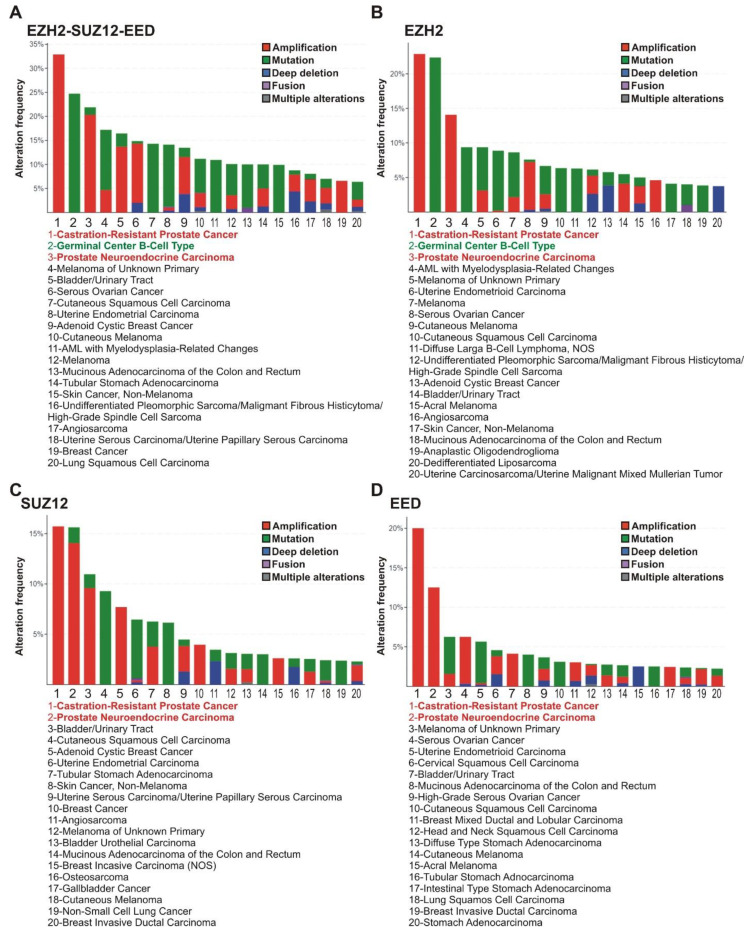
PRC2 genes are most frequently altered in subtypes of prostate and lymphoid cancers. (**A**) Alterations of any PRC2 core genes. The frequencies of alterations (gene amplifications, mutations, deep deletions, fusions or multiple alterations) of PRC2 genes in clinical samples of different cancer types were analyzed using cBioPortal. Red—CRPC and prostate neuroendocrine carcinoma; Green - GCB-DLBCL lymphoma. (**B**–**D**) Data for *EZH2*, *SUZ12* and *EED*, respectively. Please note that for some cases, the gene values for PRC2 subunits individually are lower due to co-occurrence of two or three cases of impairments (demonstrated in Supplementary File S2).

The analysis of tumor subtypes revealed a distribution of alterations in the *EZH2*, *EED,* and *SUZ12* genes (amplifications, deep deletions, mutations, fusions, and multiple alterations) in the 20 most common tumor subtypes (at least 50 samples per each subtype). The highest alteration frequency for any PRC2 genes was observed for the castration-resistant prostate cancer (CRPC; 33%), germinal center B-cell-like diffuse large B-cell lymphoma (GCB-DLBCL) (25%), and prostate neuroendocrine carcinoma (20%) (Figure 2A).

Individual examination of the *EZH2*, *SUZ12,* and *EED* genes (Figure 2B–D) showed that CRPC and prostate neuroendocrine carcinoma demonstrated an association with frequent alterations in each of the PRC2 encoding genes, while in GCB-DLBCL lymphoma a high correlation was found only with alterations in the *EZH2* gene. In accordance with tissue origin data (Figure 1), the majority of alterations in the *EZH2, SUZ12,* and *EED* genes in prostate cancer are represented by amplifications. Unlike prostate cancer, the principal *EZH2* gene abnormalities in the GCB-DLBCL are missense mutations (Figure 2).

**Figure 3:**
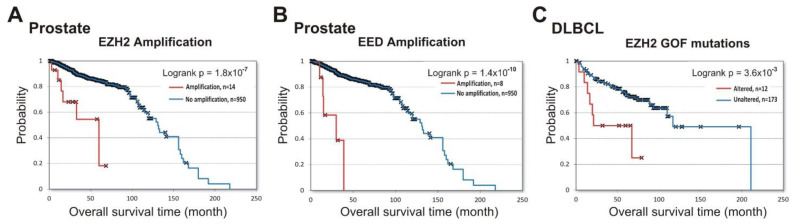
Alterations in PRC2 subunits at DNA level correlate with shorter patient survival in prostate cancer (EZH2 and EED) and DLBCL (EZH2). Analysis of correlation between alterations in DNA with patient survival was performed using cBioPortal. (**A**) Correlation between the overall survival and amplification of the *EZH2* gene in prostate cancer. The amplification (red) vs. unaltered group (blue) is shown. (**B**) Correlation between the overall survival and amplification of the *EED* gene in prostate cancer. (**C**) Correlation between the overall survival and the presence of *EZH2* GOF mutations in DLBCL. The group with *EZH2* GOF mutation (red) vs. unaltered group (blue) is shown.

Next, we queried the cBioPortal database to identify the prognostic role of PRC2 impairment in prostate cancer and in DLBCL lymphoma. Figure 3A,B show that amplifications of both *EZH2* (logrank *p* = 1.8 × 10^−7^ ) and *EED* (logrank *p* = 1.4 × 10^−10^) in prostate cancer were associated with poor prognosis (data from [33,42,43]). For the *SUZ12* gene, only two samples with amplifications and survival data are available, making the results uninterpretable.

In GCB-DLBCL, up to 21.7% of samples contained GOF missense mutations in the *EZH2* SET domain [44,45] leading to an increased PRC2 catalytic activity [46,47]. In a previous study, a high GOF frequency in GCB-DLBCL was correlated with longer patient survival [48]. However, using the available prognostic data for the DLBCL lymphoma patients from three independent studies [33,49,50], we found that GOF mutations correlate with negative overall survival (logrank *p* =3.6 × 10^−3^) (Figure 3C). These results strongly suggest that *EZH2* amplification in prostate cancer, and GOF mutations in DLBCL lymphoma, are predictors of disease progression.

### 3.2. Correlations between the EZH2, SUZ12, EED Transcription and Patient Survival

**Figure 4:**
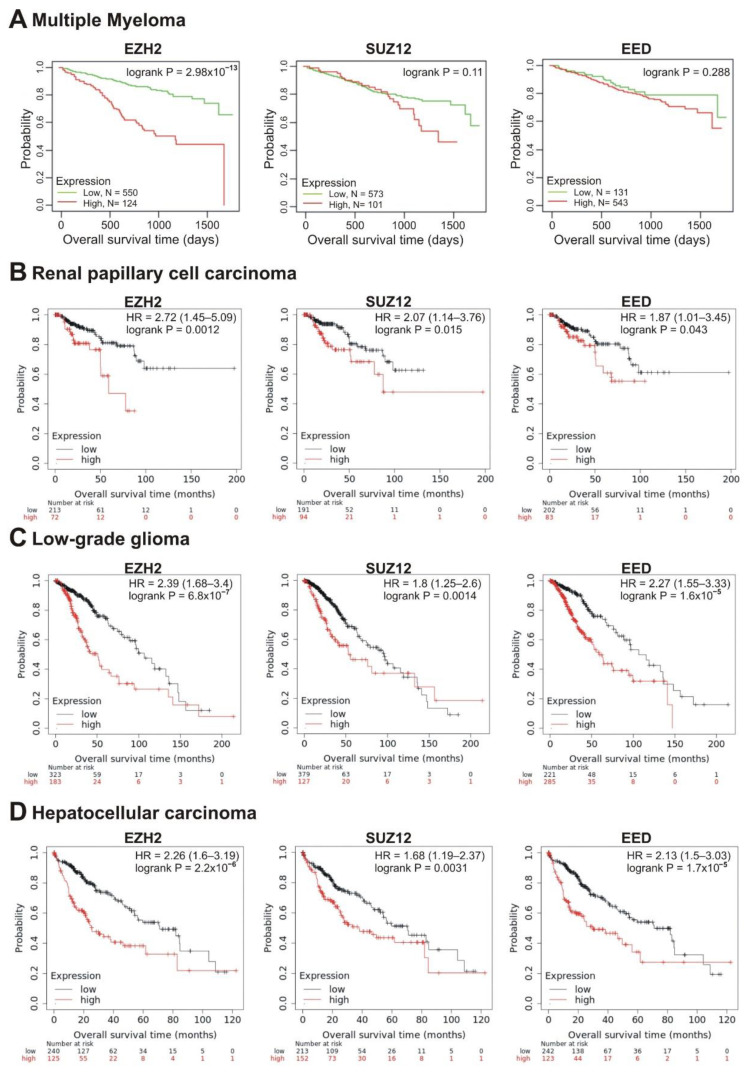
Correlation between the overall survival and the levels of *EZH2*, *SUZ12* or *EED* gene expression. (**A**) Data from the Multiple Myeloma Research Foundation CoMMpass trial (NCT01454297; version IA11a). The patients were divided into two cohorts—with low (green) or high (red) levels of gene expression. (**B**–**D**) The analysis was performed using the KMplot resource for renal papillary cell carcinoma (**B**), low-grade glioma (**C**) and hepatocellular carcinoma (**D**). The cohort with low level of gene expression is colored in black, cohort with high expression is colored in red.

We next investigated the correlation of transcription of the *EZH2*, *SUZ12* and *EED* genes with patient prognosis. Investigating the survival prognosis in multiple myeloma (MM) patients, we have shown that PRC2 core genes, *EZH2, SUZ12* and *EED*, were significantly overexpressed in MM cells compared to normal plasma cells, making these cells sensitive to EPZ-6438, an inhibitor of EZH2 [51]. Moreover, high *EZH2* expression correlated with poor prognosis in MM [52]. Herein, we studied the correlation of the elevated transcription of PRC2 core genes with the survival rate. RNAseq data were obtained from 674 newly diagnosed MM patients with longitudinal follow-up (Multiple Myeloma Research Foundation CoMMpass trial; NCT01454297; version IA11a). Patients were divided into two cohorts based on high (Figure 4A, red lines) or low (Figure 4A, green lines) mRNA levels of PRC2 subunits. The life expectancy (overall survival; OS) was analyzed for these two groups. Figure 4A demonstrates that the increased EZH2 transcription was significantly (logrank *p* = 2.98 × 10^−13^) correlated with poor prognosis. At the same time, high abundance of *SUZ12* and *EED* transcripts showed no significant correlation with survival, suggesting that *EZH2* expression is the strongest PRC2 prognostic marker in MM.

To analyze the correlations of *EZH2*, *SUZ12* and *EED* expression with the survival of patients with other tumors, we used RNAseq data in the Pan-Cancer dataset of the Kaplan-Maier plotter website (www.kmplot.com, accessed on 15 January 2021). The results are summarized in Table 1.

In total, in 10 tumor types, the higher expression of at least one of the *EZH2*, *SUZ12,* or *EED* genes was significantly correlated with poor prognosis (logrank *p* < 0.05) (Table 1). In renal papillary cell carcinoma, low-grade glioma and hepatocellular carcinoma, poor prognosis was significantly correlated with high expression of all three genes, *EZH2*, *SUZ12,* and *EED* (Table 1, Figure 4B–D).

**Figure 5:**
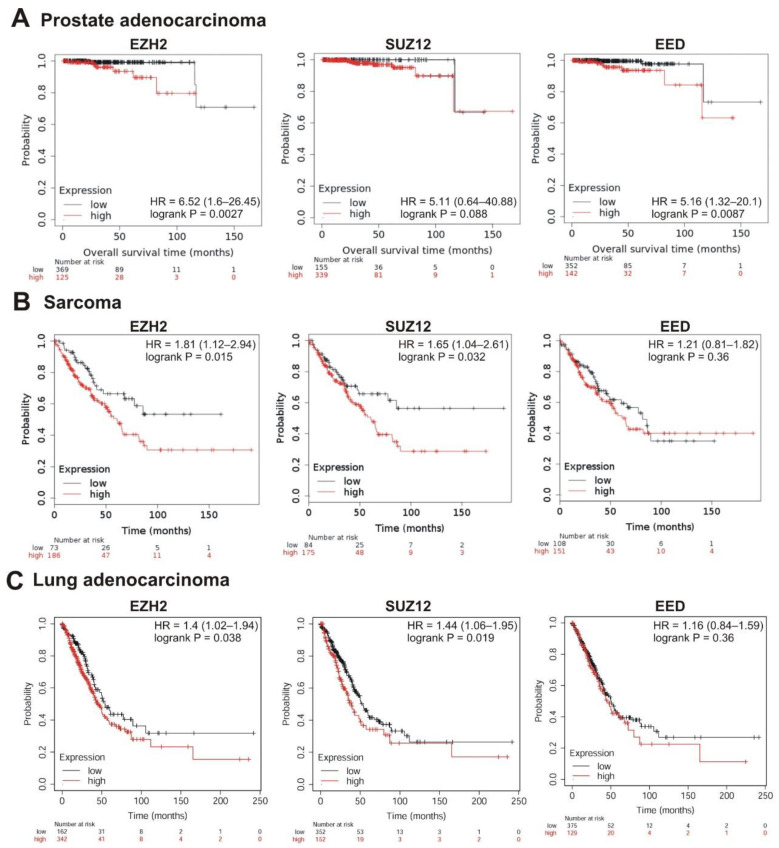
Correlation between the overall survival and the levels of *EZH2*, *SUZ12* or *EED* gene expression. (**A**) Prostate adenocarcinoma, (**B**) sarcoma, and (**C**) lung adenocarcinoma. The analysis was performed using KMplot resource.

**Figure 6:**
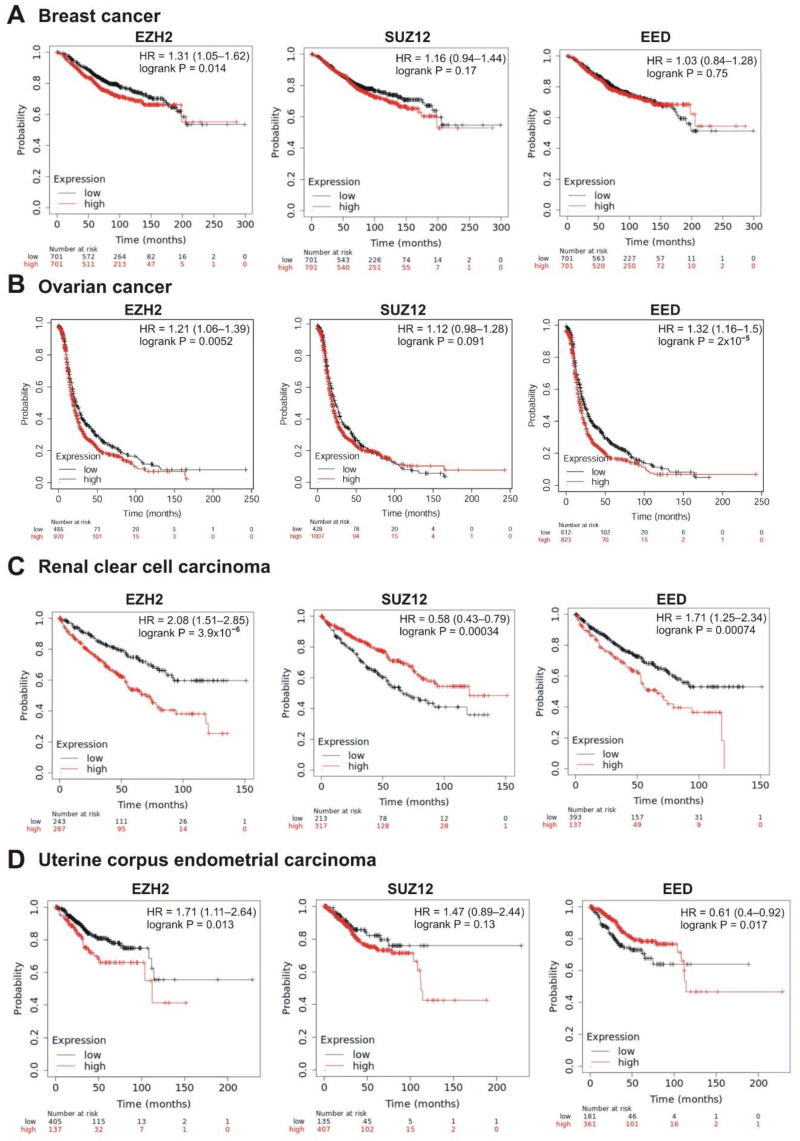
Correlation between the overall survival and the levels of *EZH2*, *SUZ12* or *EED* gene expression. (**A**) Breast cancer, (**B**) ovarian cancer, (**C**) renal clear cell carcinoma, and (**D**) uterine corpus endometrial carcinoma.

The strongest correlation between high mRNA abundance and poor survival was observed for *EZH2* and *EED* in prostate adenocarcinoma (logrank *p* < 0.01; hazard ratio 6.52 and 5.16, respectively). The high expression of *SUZ12* in this tumor also marked a tendency to poor prognosis (hazard ratio = 5.11, logrank *p* = 0.088) (Table 1, Figure 5A). A significant correlation with poor prognosis was also observed for high *EZH2* and *SUZ12* expression in sarcoma (Table 1, Figure 5B) and in lung adenocarcinoma (Table 1, Figure 5C); for *EZH2* in breast cancer (Table 1, Figure 6A); for *EZH2* and *EED* expression in ovarian cancer (Table 1, Figure 6B).

Intriguingly, in several cases, higher expression of PRC2 genes was differently but significantly correlated with patient survival. In renal clear cell carcinoma, higher expression of *EZH2* and *EED* was significantly correlated with poor prognosis, whereas high levels of *SUZ12* were associated with longer survival (Table 1, Figure 6C). For the uterine corpus endometrial carcinoma, high expression of *EZH2* was correlated with poor survival, although higher expression of *EED* was associated with a longer survival (Table 1, Figure 6D).

Remarkably, opposite correlations were observed for *EZH2*, *SUZ12,* and *EED* in some cancers, with higher expression being associated with a longer survival, suggesting a tumor suppressor role for PRC2 in those cases. In particular, the higher expression of either of the PRC2 genes correlated with a longer patient survival in gastric cancer and thymoma (Table 1, Supplementary File S3).

### 3.3. Dependency of Tumor Cell Lines on EZH2, SUZ12, and EED

An alternative approach to identifying PRC2-dependent tumors is the screening of a large panel of cell lines of different tissues-of-origin for growth inhibition upon suppression of PRC2 function. Currently, an online project called DepMap (www.depmap.org, accessed on 6 October 2020) allows one to predict and approximate the results of such screening [34,35]. In DepMap, a comprehensive library of human genes has been knocked down by RNAi or, more recently, knocked out through CRISPR technology in large panels of human cell lines representing different tumor types. The probability of dependency of each cell line on the queried gene is represented as the dependency scores, where strong negative values mark the cases where a given gene is especially important for growth or survival of the respective cell lines. The cell line is considered dependent if the probability of dependency is >0.5; a score of 0 is equivalent to a non-essential gene, whereas a score of −1 corresponds to the median of all common essential genes, respectively. For gene effects, a score of <−0.5 indicates sensitive cell lines (depletion of most cells), while <−1 represents strong killing.

**Figure 7:**
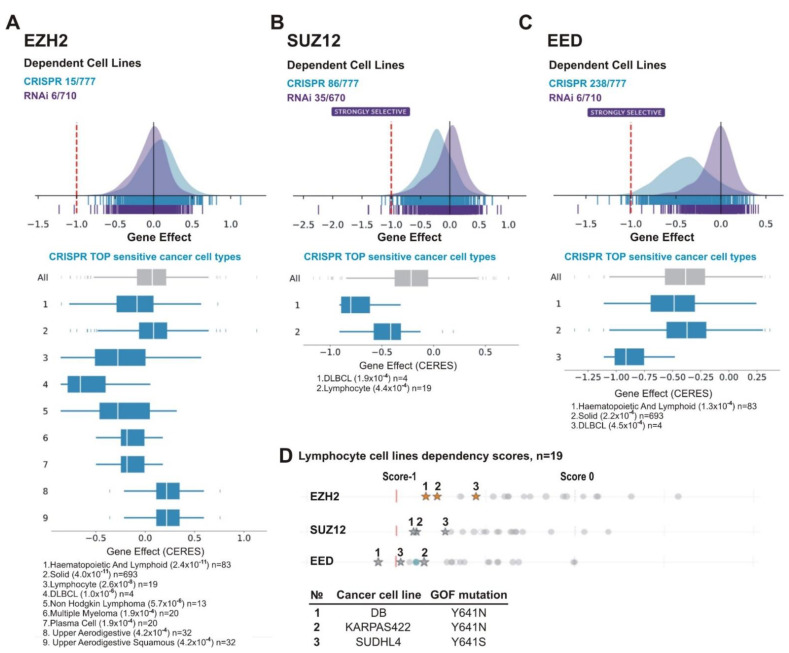
Lymphoid cancer cell lines are particularly sensitive to *PRC2* knockout. The analysis was performed using DepMap database. (**A**) *EZH2*, (**B**) *SUZ12* and (**C**) *EED*. (**A**–**C**) UP: schematic representation of the effects of gene knockout (by CRISPR, blue) or knockdown (by RNAi, purple) on cells growth and viability. X-axis: dependency scores (Gene Effect) reflect the dependence of cell growth and survival upon depletion of a particular gene. Negative values indicate that cell proliferation is decreasing upon the gene depletion and score less than −0.5 indicates that the gene is required for survival of a given cancer cell line. Numbers next to “CRISPR” and “RNAi” indicate the amount of sensitive cell lines with score <−0.5/total number of cell lines in each case. DOWN: The TOP sensitive cancer cell types to knockout of *EZH2* (**A**), *SUZ12* (**B**), and *EED* (**C**) by CRISPR are shown. (**D**) Lymphoma lines carrying EZH2 GOF mutations are in TOP of cell lines sensitive to *EZH2*, *SUZ12* or *EED* knockout. UP: The dependency scores for a panel of lymphoma lines upon knockout of PRC2 genes by CRISPR. Asterisks indicate the cell lines with GOF mutations in the EZH2 SET domain, grey circles—lines without GOF mutations. DOWN: The GOF mutations in test cell lines are indicated.

Figure 7A–C demonstrates that, for all PRC2 subunits, CRISPR knockout showed a stronger inhibitory effect on cell growth than RNAi knockdown. Figure 7B,C shows that upon CRISPR knockout the SUZ12 and EED genes appeared as ‘strongly selective’ and were important for growth/survival of 86/777 and 238/777 cell lines, respectively. For EZH2, only 15/777 lines were sensitive (Figure 7A). One factor of the differential roles of individual PRC2 subunits is the EZH2 paralog protein EZH1 (Enhancer of Zeste Homolog 1). This paralog has much lower methyltransferase activity [79] and is less important for development [80]. However, EZH1 can partially replace EZH2 in cells where EZH2 expression is impaired [81,82,83]. Consistent with this possibility, there are no homologs for the SUZ12 and EED proteins in humans. Due to a higher sensitivity of cell lines upon gene knockout, further analysis was based on CRISPR/Cas9 data.

We thus queried the DepMap CRISPR *EZH2, SUZ12,* or *EED* knockout data for Top Co-dependency Pearson correlations. In the case of each gene knockout (*EZH2*, *SUZ12,* or *EED*), two other subunits were identified as Top co-dependent (Table 2). This is in accordance with their cooperative function in regulation of transcription. Thus, DepMap corroborates biochemical data indicating the validity of this analysis.

We next analyzed cell lines of various tissue origin. Among the most sensitive types for each of the PRC2 gene knockouts were lymphoma cell lines including DLBCL (Figure 7A–C). This is consistent with findings suggesting that lymphoma cells, in particular, DLBCL, are sensitive to *EZH2* depletion and *EZH2* inhibitors [84,85,86,87]. Because this malignancy is frequently characterized by GOF missense mutations of the *EZH2* SET domain [44,45,88], we focused on lymphocyte cell lines. The panel in the DepMap project includes three DLBCL cell lines with *EZH2* GOF mutations: DB (Y641N), KARPAS422 (Y641N) and SUDHL4 (Y641S). Noteworthy, these cell lines are among the most sensitive to knockouts of each of PRC2 subunits (Figure 7D), with DB being among the Top10 of the sensitive lines upon depletion of any of PRC2 subunit (Table 3). This suggests that GOF mutations in the SET domain of EZH2 can augment the sensitivity of lymphoma cells to the inhibition of either *EZH2, SUZ12,* or *EED*.

Along with lymphoma, the cell lines of other tissue origins were among the most sensitive to PRC2 knockouts (Table 3, Supplementary File S1). These included TUHR10TKB kidney cancer cell line sensitive to depletion of either of the PRC2 subunits (Table 3); the OC316 ovarian cancer line that was in the Top10 for *EZH2* and *EED* (Table 3) and in the Top40 for *SUZ12* (Supplementary File S1) and, finally, the VCAP prostate cancer line—in the Top10 for *EZH2* and *SUZ12* (Table 3) and in the Top40 for *EED* (Supplementary File S1).

Recent data retrieved a few genetic markers associated with sensitivity of tumor cells to PRC2 inhibition. The epithelioid sarcoma (for which tazemetostat has been approved by the FDA [26,27,28]) is deficient in the SMARCB1 (also known as INI1 or SNF5) subunit of the SWI/SNF family [27]. Several other SMARCB1 mutant cancers, including malignant rhabdoid tumors (MRT) and atypical teratoid rhabdoid tumors (ATRT) are candidates for treatment with EZH2 inhibitors [89,90]. Several components of the SWI/SNF complex (SMARCA2, SMARCA4, ARID1A, PBRM1) are also promising targets for the PRC2 inhibitor therapy. Preclinical and Phase 1 studies suggest an efficacy of tazemetostat in the treatment of SCCOHT (small cell carcinoma of the ovary hypercalcaemic type) deficient in SMARCA4 (also known as BRG1) and SMARCA2 (a.k.a. BRM) [91]. In addition, cell lines carrying ARID1A- and PBRM1 mutants are sensitive to EZH2 inhibition [92,93,94].

With this information, we aimed to identify more gene candidates as markers for sensitivity to PRC2 inhibition. In so doing, we generated a Python application to challenge a hypothesis of dependence between the growth inhibition upon knockout of *EZH2*, *SUZ12*, or *EED*, and the presence of each documented mutation (https://github.com/genesolution/PRC2_data, uploaded on 25 March 2021). For the test hypothesis, a cell line was considered to be “sensitive” if it had a score <−0.5 (inhibition of the majority of cells). This value was provided to us as the universal effect limit for the DepMap tables due to the normalization that had been previously done by the DepMap table authors. In our analysis, we used all types of genetic alterations, except synonymous mutations marked as “silent” (using Variant_Classification column).

The level of *p*-value significance was set at *p* < 0.05. All mutations for which the test hypothesis was found to be significant (*p* < 0.05) are listed in Supplementary File S4 “PRC2 significant *p*-value_no_silent EZH2, SUZ12, EED”. The number of some gene mutations is very low (there are initially only a few of them, or most are classified as “Silent”). In this case, it is inappropriate to use the *p*-value to draw a reliable conclusion about their effect. However, we kept all the obtained values to indicate the possibly promising genes for further analysis. For the detailed analysis, the cases with the mutation present in at least 10 cell lines were suggested as statistically relevant.

**Figure 8:**
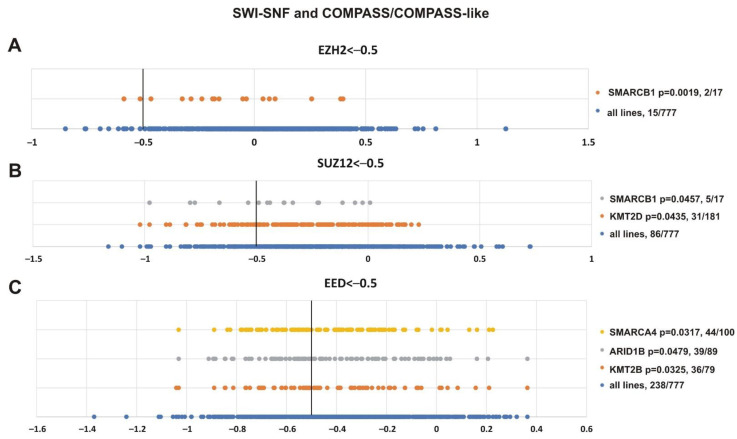
Mutations in genes encoding subunits of the SWI/SNF and COMPASS-like complexes correlate with sensitivity to PRC2 knockout. (**A**) *EZH2*, (**B**) *SUZ12* and (**C**) *EED.* The X-axis shows the dependency scores of the lines with alterations of a particular gene (orange, grey and yellow circles) vs. all cell lines (blue circles, 777 in total). Black bold vertical line indicates the dependency score −0.5 (the score <−0.5 mean that gene is critical for cells survival). *p*-value—the statistical significancy for hypothesis that the cell lines with SWI/SNF or COMPASS mutations will have a score of less than −0.5. The number of cancer cell lines bearing an altered gene with a score <−0.5/total number of cell lines bearing the altered gene is indicated at the right.

To validate the analysis, we first tested the presence of SMARCB1 and other SWI/SNF subunits in the resulting list (Figure 8). Additionally, we paid attention to mutations of transcriptional activators from the Trithorax group, i.e., the subunits of COMPASS- and COMPASS-like complexes that antagonize PcG activity [2,4,5,95]. Figure 8 shows that SMARCB1 alteration was correlated with the sensitivity of cell lines to *EZH2* (*p* = 0.0019) and *SUZ12* (*p* = 0.0457) knockdown. Impairment of two SWI/SNF subunits, SMARCA4 (*p* = 0.0317) and ARID1B (*p* = 0.0479), was correlated with the sensitivity to *EED* knockout. Furthermore, among COMPASS/COMPASS-like complexes, depletion of KMT2D (*p* = 0.0435) and KMT2B (*p* = 0.0325) histone methyltransferases correlated with knockouts of *SUZ12* and *EED*, respectively.

**Figure 9:**
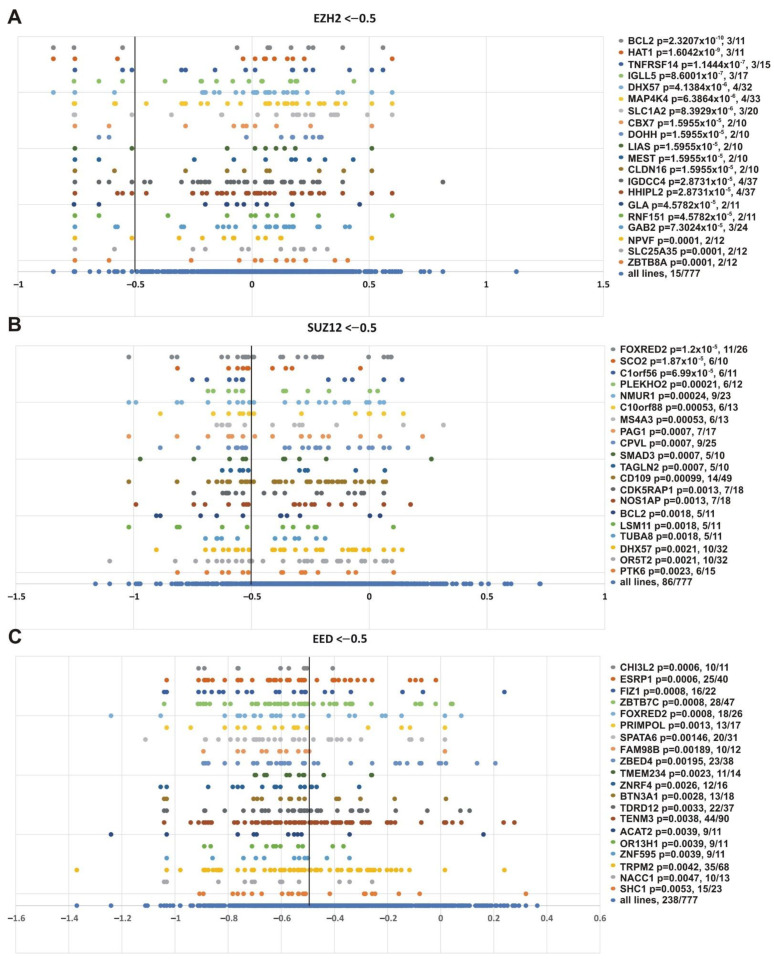
Top 20 genes showing the best correlation with the sensitivity of cancer cell lines to PRC2 genes knockout. (**A**) *EZH2*, (**B**) *SUZ12* and (**C**) *EED.* Other designations as in Figure 8.

While *SMARCB1* was in the resulting list, this marker was not among the top correlated genes. Figure 9 shows the genes whose impairment demonstrated the best correlation with the sensitivity of cell lines to knockouts of *EZH2* (Figure 9A), *SUZ12* (Figure 9B) and *EED* (Figure 9C).

This analysis produced several interesting observations. First, some genes with the lowest *p-*values are known to be functionally connected to PcG or TrxG systems. CPVL (carboxypeptidase, vitellogenic-like), which was significant in *SUZ12* knockout (*p* = 0.0007), can be co-purified with the Polycomb repressive complex 1 (PRC1) subunits PCGF2 and PHC2 in affinity-capture MS [96]. SMAD3 was significant for *SUZ1*2 knockout (*p* = 0.0007); it is an important component of the transforming growth factor β (TGFβ) signaling, and interacts with the BRG1 SWI/SNF complex (subunits SMARCA4, ARID1B/BAF250b, SMARCC2/BAF170, and SMARCC1/BAF155) [97] and CREBBP [97,98,99]. ESRP1 (epithelial splicing regulatory protein 1) and FAM98B (family with sequence similarity 98, member B) were significant in *EED* knockout (*p* = 0.0006 and *p* = 0.00189); they are minor components of cPRC1 purified via BMI1 (PCGF4) [100]. Among the markers sensitive to *EZH2* knockout, CBX7 (*EZH2*, *p* = 1.5955 × 10^−5^) is a subunit of PRC1 [96], and ZBTB8A (*EZH2*, *p* = 0.0001) interacts with CBX8 in two hybrid screening [101]. IGLL5 (immunoglobulin lambda-like polypeptide 5), significant for *EZH2* (*p* = 8.6001 × 10^−7^), is a minor component of PRC2 purified via EZH2 or SUZ12, and of PRC1 purified with antibodies against RNF2/RING1B [100].

Second, several genes were correlated with knockouts of several PRC2 subunits (Figure 9 and Supplementary File S4). This group is of special interest, since different PRC2 subunits are expected to function together and all are required for the PRC2 catalytic activity. In particular, the TOP list contains *FOXRED2* gene impairment, which showed the best correlation dependency with *SUZ12* knockout (*p* = 1.2 × 10^−5^) and correlated with *EED* knockout (*p* = 0.0008). BCL2 showed the best correlation with *EZH2* knockout (*p* = 2.3207 × 10^−10^) and correlated with *SUZ12* (*p* = 0.0018) knockout. The *DHX57* gene was correlated with *SUZ12* (*p* = 0.0021) and *EZH2* (*p* = 4.1384 × 10^−6^ knockout, respectively. The *DHX57* gene has been attributed to the PcG system, since DHX57 interacts with CBX2 in two hybrid screening [101,102].

**Figure 10:**
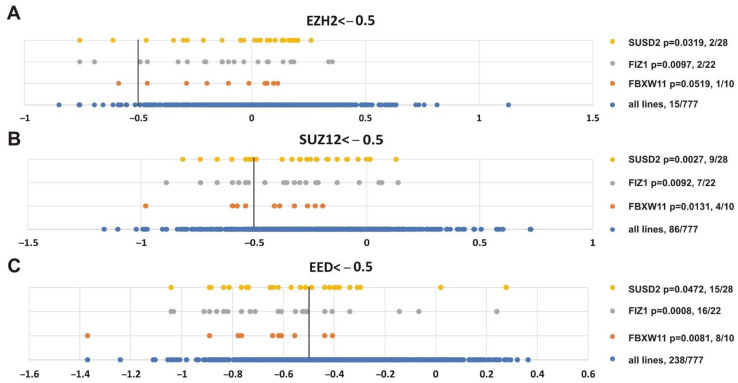
Genes showing significant correlation with the sensitivity of cancer cell lines to knockout of any PRC2 subunit. (**A**) *EZH2*, (**B**) *SUZ12* or (**C**) *EED*. Other designations as in Figure 8.

Finally, we were interested in finding genes whose impairment was correlated with inhibition of the three PRC2 subunits. Figure 10 shows that alterations in *SUSD2* (sushi domain containing 2), *FIZ1* (FLT3 interacting zinc finger 1) and *FBXW11* (F-box and WD repeat domain containing 11) genes correlated with knockout of *EZH2* (*p* = 0.0319, *p* = 0.0097, *p* = 0.0519, respectively), *SUZ12* (*p* = 0.0027, *p* = 0.0092, *p* = 0.0131, respectively) and *EED* (*p* = 0.0472, *p* = 0.0008, *p* = 0.0081, respectively). Intriguingly, FBXW11 co-purifies with EED [103], SUZ12 and EZH2 [104].

## 4. Discussion

The PRC2 repressor complex plays a central role in maintaining the correct pattern of gene expression in multicellular organisms. Dysfunction of this complex is associated with many pathologies, including cancer. The development of small-molecule PRC2 inhibitors requires the identification of types and subtypes of tumors that are dependent on the PRC2 function. Current data are largely focused on the impairment of the EZH2 methyltransferase, a core PRC2 subunit, in cancer. Two other core PRC2 subunits, SUZ12 and EED, have been less studied. Here, we analyzed the role of all core PRC2 subunits in a series of clinical tumor samples (cBioPortal and Kaplan-Maier plotter resources) and in a panel of tumor cell lines (assayed in the DepMap project).

The analysis of cBioPortal clinical data indicates that amplification of *EZH2*, *SUZ12,* and *EED* genes was not limited to one particular malignancy but varied between different tumor types. The ovarian, skin, prostate and soft tissue tumors are characterized by over 1% amplification frequency of *EZH2* and *EED* genes with the highest frequency in ovarian cancer: 11.4% and 9.84%, respectively. The *SUZ12* gene was amplified in >1% of breast, esophagus/stomach, and bladder/urinary tract tumors where its amplification correlated with >1% of *EED* amplification. In addition, over 1% of *EED* amplification was observed in the head and neck cancer specimens.

Detailed analysis of tumor subtypes identified CRPC and prostate neuroendocrine carcinoma as the tumors with the highest amplification rate (12–23%) of *EZH2*, *SUZ12* and *EED* genes. The amplification of *EZH2* in prostate cancer was first shown by Saramaki et al. [39] where it was significantly (*p* < 0.05) correlated with increased EZH2 protein levels. Subsequent studies of prostate cancer demonstrated that high *EZH2* and *SUZ12* expression correlate with metastasis progression [60], and for *EZH2^high^*, with poor survival prognosis [58,59]. Here, we show that amplification and higher expression of *EZH2* and *EED* correlate with poor survival, further supporting the involvement of *EZH2* and its PRC2 protein partners in prostate cancer.

Missense mutations of *EZH2*, but not *SUZ12* or *EED*, were most frequent in GCB-DLBCL (over 20%, cBioPortal clinical data). Missense mutations in GCB-DLBCL are frequently represented by GOF mutations in the EZH2 SET domain [44,45] that enhance PRC2 methyltransferase activity, leading to an abnormally high level of the chromatin repressive H3K27me3 mark [46,47]. The survival analysis based on three independent studies [33,49,50] indicates that GOF mutations in *EZH2* correlate with poor prognosis in DLBCL. Remarkably, the DLBCL cell lines (DepMap project) with GOF mutations in *EZH2* were among the most sensitive to PRC2 knockout.

The high *EZH2*, *SUZ12,* and *EED* expression in tumor samples has been attributed to the decreased patient survival in several tumor types [2,19,20]. Here we demonstrated that along with prostate adenocarcinoma, other tumor types show a correlation between high *EZH2*, *SUZ12* and *EED* levels and a shorter survival, suggesting that these cancers could be potential targets for *EZH2*/*SUZ12*/*EED* inhibitor therapy. Importantly, the high expression of either of the PRC2 subunits correlate with poor prognosis in patients with renal papillary cell carcinoma, low-grade glioma and hepatocellular carcinoma. Furthermore, poor prognosis was revealed for sarcoma and lung adenocarcinomas (*EZH2*^high^ and *SUZ12*^high^); ovarian, renal clear cell carcinomas (*EZH2*^high^ and *EED*^high^); and for MM, as well as breast and endometrial carcinomas (*EZH2*^high^).

The *EZH2* sensitivity to inhibitor therapy was shown to be dependent on the presence of mutations in secondary genes. In particular, the SWI/SNF subunit SMARCB1 has been approved as an effective marker of metastatic or locally advanced epithelioid sarcoma sensitivity to tazemetostat [26,27,28], while SMARCA2, SMARCA4, ARID1A, and PBRM1 are potential marker candidates [91,92,93,94,105]. The analysis of correlation between impairment of individual genes and the sensitivity of 777 cell lines to knockout of *EZH2*, *SUZ12,* or *EED* genes allowed us to predict new gene mutations as tentative markers for PRC2 inhibitor therapy. The list of these genes includes the genes encoding SWI/SNF complex subunits, namely, *SMARCB1*, *SMARCA4*, and *ARID1B*. Furthermore, impairment of *KMT2D* and *KMT2B* COMPASS-like genes was also significant. Both SWI/SNF and COMPASS-like are the Trithorax group complexes that counteract the repressive activity of PRC2 suggesting that SWI/SNF and COMPASS-like members can be directly involved in the regulation of PRC2 mediated carcinogenesis. Accordingly, loss-of-function mutations in *KMT2D* can occur in B-cell lymphomas together with GOF mutations of the *EZH2* gene [106]. Moreover, similarly to *EZH2* GOF, the loss of *KMT2D* promotes lymphomagenesis [107,108].

A significant overlap between DNA methylation and H3K27me3 binding in EZH2 inhibitor target genes was described in MM in association with resistance to the EZH2 inhibitor [51]. These two repressive marks have been shown to be mechanistically linked [109]. Of interest, the addition of a low dose of DNMT inhibitor can resensitise EZH2 inhibitor-resistant MM cells to EZH2 inhibition [51], suggesting that combination of EZH2 and DNMT inhibitors could be of therapeutic interest.

Impairment of several newly identified genes showed even higher correlations with sensitivity of tumor cell lines to PRC2 knockout. Alterations of *BCL2* (B-cell CLL/lymphoma 2) showed the best correlation with *EZH2* and correlated significantly with *SUZ12* knockout. Alterations in *BCL2* frequently co-occur with *EZH2* in DLBCL, and combined inhibition of BCL2 and EZH2 has been considered a rational therapeutic approach [110].

Several other factors, including CPVL, SMAD3, CBX7, ESRP1, FAM98B, IGLL5, DHX57, and FBXW11 were shown to be physically connected to PcG or TrxG systems. Among these factors SMAD3 (significant in *SUZ1*2 knockout) is an important component of TGFβ signaling. In response to TGFβ SMAD3 interacts with and is acetylated by histone acetyltransferase CREBBP [97,98,99]. SMAD3 acetylation has been shown to be critical for potentially oncogenic Epstein-Barr virus lytic program [99]. Moreover, the recruitment of RbBP5 component of COMPASS-like complexes and the formation of H3K4me3 at SNAIL transcription start site during epithelial-mesenchymal transition are dependent on SMAD3 and CBP in the DU145 prostate cancer cell line [111].

Finally, we note that, while higher expression of PRC2 components is frequently associated with poor prognosis, in a significant number of cases, the opposite correlation was observed. This is consistent with previous reports identifying inactivating PRC2 mutations in hematological cancers, indicating that PRC2 can play a context-dependent tumor suppressor role. EZH2 inactivating mutations have been reported in myeloid hemopathies including chronic myelomonocytic leukemia (CML), myelofibrosis, myelodysplastic syndrome (MDS) and AML [112,113,114]. Furthermore, EZH2-loss-of-function mutations were associated with a poor outcome in MDS, CML and myelofibrosis [112,113,115]. EZH2-loss of function mutations can also contribute to the development of T-ALL [116,117]. It has been shown that PRC2 inhibits the self-renewal potential of immature lymphoid progenitors [118,119,120], whereas it stimulates mature cell proliferation [51,52,121,122,123]. Therefore, PRC2 may act as an oncogenic factor in mature hematopoietic cells while exerting tumor suppressor activities in undifferentiated or immature cells. While these tumors might be insensitive to EZH2 inhibitors, a correlation between higher expression of PRC2 and good prognosis might be of interest as it might suggest a possible sensitivity of these tumors to inhibitors of KDM6 that demethylate H3K27me3 [124], or of active chromatin components, such as those previously reported for SUZ12 inactivating mutations that sensitize several cancer types to inhibitors of Bromodomain and Extra-Terminal motif (BET) proteins, a family of proteins that counteract gene silencing with a chromatin activation function [125].

## 5. Conclusions

In summary, we performed bioinformatic analysis of genomic and transcriptomic data across multiple tumor types to identify clinically significant trends related to PRC2. With this analysis, we identified new tumors that could benefit from targeted PRC2 treatment. Moreover, DepMap interactive genes could present new potential markers of tumors that are more sensitive to PRC2 inhibitors. The next step would be to elucidate the functional role of identified genes in PRC2 oncogenic function and sensitivity to inhibitors directly. Furthermore, studies are needed to assess the cellular and molecular effects of PRC2 inhibitors. For instance, while PRC2 is frequently overexpressed in breast cancer, single-cell analysis suggests that drug-resistant cells may downregulate H3K27me3 at many loci [126]. Our analysis makes it possible to filter individual genetic factors related to tumor sensitivity to PRC2 inhibition. These factors could set the stage for the selection of patients for individualized treatment with PRC2-targeting drugs.

## Figures and Tables

**Table 1:** Correlations of *EZH2*, *SUZ12* and *EED* gene expression with overall survival in different tumor types. The hazard ratio (HR) and *p-*values are shown in red when higher gene expression is correlated with a shorter survival, and in blue in case of a longer survival. logrank *p* < 0.05 are boldfaced. The “Refs to poor prognosis” columns indicate references to previous studies showing the correlation with poor survival of the respective PRC2 subunit.

Cancer Type	№ of Samples	EZH2		Refs to Poor Prognosis	SUZ12		Refs to Poor Prognosis	EED		Refs to Poor Prognosis
		*HR*	Logrank *p*		*HR*	Logrank *p*		*HR*	Logrank *p*	
**Statistically significant correlation with poor survival upon higher expression of any PRC2 subunits**										
Renal papillary cell carcinoma	285	**2.72**	**0.0012**	[53,54,55]	**2.07**	**0.015**	ND	**1.87**	**0.043**	ND
Low grade glioma	506	**2.39**	**6.8 × 10^−7^ **	ND	**1.8**	**0.0014**	ND	**2.27**	**1.6 × 10^−5^ **	ND
Hepatocellular carcinoma	365	**2.26**	**2.2 × 10^−6^ **	[56]	**1.68**	**0.0031**	[57]	**2.13**	**1.7 × 10^−5^ **	ND
**Statistically significant correlation upon higher expression of some of PRC2 subunits**										
Prostate adenocarcinoma	494	**6.52**	**0.0027**	[58,59,60]	5.11	0.088	[60]	**5.16**	**0.0087**	ND
Sarcoma	259	**1.81**	**0.015**	[61,62]	**1.65**	**0.032**	[61]	1.21	0.36	[61]
Lung adenocarcinoma	504	**1.4**	**0.038**	NSCLC [63,64,65,66]	**1.44**	**0.019**	ND	1.16	0.36	ND
Breast cancer	1402	**1.31**	**0.014**	[67,68,69]	1.16	0.17	ND	1.03	0.75	ND
Ovarian cancer	1435	**1.21**	**0.0052**	[70]	1.12	0.091	[70]	**1.32**	**2 × 10^−5^ **	ND
**Opposite statistically significant correlation with survival**										
Renal clear cell carcinoma	530	**2.08**	**3.9 × 10^−6^ **	[53,54,55]	**0.58**	**0.00034**	ND	**1.71**	**0.00074**	ND
Uterine corpus endometrial carcinoma	542	**1.71**	**0.013**	ND	1.47	0.13	ND	**0.61**	**0.017**	ND
**Statistically significant correlation with positive prognosis upon higher expression of any PRC2 subunits**										
Gastric cancer	873	**0.75**	**0.0015**	[71,72]	**0.65**	**3.5 × 10^−7^ **	[73]	**0.57**	**3.8 × 10^−9^ **	ND
Thymoma	118	**0.13**	**0.0012**	ND	**0.07**	**0.00076**	ND	**0.25**	**0.059**	ND
**Other cancers analyzed**										
Acute myeloid leukemia	132	**0.5**	**0.0029**	ND	0.77	0.25	ND	0.78	0.26	ND
Lung squamous cell carcinoma	494	**0.68**	**0.017**	NSCLC [63,64,65,66]	**0.75**	**0.047**	ND	0.8	0.16	ND
Head-neck squamous cell carcinoma (HNSCC)	499	**0.61**	**0.0039**	[74]	0.79	0.15	[75]	**0.76**	**0.043**	ND
Stomach adenocarcinoma	354	**0.57**	**0.00071**	ND	0.83	0.28	ND	0.81	0.22	ND
Thyroid carcinoma	501	**0.54**	**0.23**	[76]	2.87	0.056	ND	0.43	0.083	ND
Cutaneous melanoma	458	1.15	0.3	[58]	**0.55**	**6.1 × 10^−5^ **	ND	**0.6**	**0.00039**	ND
Rectum adenocarcinoma	159	0.57	0.19	ND	**0.43**	**0.034**	ND	**0.31**	**0.014**	ND
Esophageal Squamous Cell Carcinoma	81	0.62	0.24	ND	**0.28**	**0.017**	ND	**0.37**	**0.015**	ND
Bladder Carcinoma	404	0.76	0.063	[77]	1.28	0.13	[77,78]	**0.51**	**7 × 10^−6^ **	ND
Glioblastoma	152	1.35	0.16	ND	0.8	0.31	ND	0.78	0.17	ND

**Table 2:** The Top Co-dependency Pearson correlations upon knockout of PRC2 components by CRISPR/Cas9 technology in tumor cell lines (DepMap project data). Bold—subunits of PRC2 complex.

	Top 5 Co-Dependencies upon EZH2 Deletion			Top 5 Co-Dependencies upon SUZ12 Deletion			Top 5 Co-Dependencies upon EED Deletion	
Rank	Gene	Pearson Correlation	Rank	Gene	Pearson Correlation	Rank	Gene	Pearson Correlation
**1**	**EED**	**0.67**	**1**	**EED**	**0.64**	**1**	**EZH2**	**0.67**
**2**	**SUZ12**	**0.61**	**2**	**EZH2**	**0.61**	**2**	**SUZ12**	**0.64**
3	DOT1L	0.41	3	RING1	0.37	3	DOT1L	0.40
4	RING1	0.36	4	PCGF1	0.32	4	RING1	0.36
5	PCGF1	0.33	5	DOT1L	0.29	5	MEN1	0.35

**Table 3:** Top cell lines most sensitive to *EZH2*, *SUZ12,* and *EED* knockouts by CRISPR/Cas9. Red—cancer cell lines present in TOP15 sensitive upon depletion of any PRC2 subunit; purple—present in case of depletion of any of two of PRC2 subunits.

	*EZH2*		*SUZ12*		*EED*	
	Cell Line Name, Primary Disease	Dependency Score	Cell Line Name, Primary Disease	Dependency Score	Cell Line Name, Primary Disease	Dependency Score
1	DB, Lymphoma, DLBCL	−0.848	SUM52PE, Breast Cancer	−1.162041582	TUHR10TKB, Kidney Cancer	−1.369846119
2	KARPAS422, Lymphoma, DLBCL	−0.75998	SNU216, Gastric Cancer	−1.101402111	TE8, Esophageal Cancer	−1.240713197
3	MUTZ8, Leukemia, AML	−0.75646	SKBR3, Breast Cancer	−1.020127652	L33, Pancreatic Cancer	−1.111219428
4	OC316, Ovarian Cancer	−0.75617	VCAP, Prostate Cancer	−0.990346606	DB, Lymphoma, DLBCL	−1.103548203
5	MERO14, Lung Cancer	−0.69188	KU812, Leukemia, CML	−0.982683494	SKBR3, Breast Cancer	−1.054003392
6	UHO1, Lymphoma, Hodgkins	−0.65357	TUHR10TKB, Kidney Cancer	−0.977821458	OC316, Ovarian Cancer	−1.040763201
7	SMZ1, Lymphoma, unspecified	−0.61017	CL11, Colon/Colorectal Cancer	−0.972156719	EN, Endometrial/ Uterine Cancer	−1.031582739
8	U2OS, Bone Cancer	−0.58734	DB, Lymphoma, DLBCL	−0.903542458	U2OS, Bone Cancer	−1.018695817
9	TUHR10TKB, Kidney Cancer	−0.58528	SKMM2, Myeloma	−0.887244551	EMTOKA, Endometrial/ Uterine Cancer	−1.006987228
10	VCAP, Prostate Cancer	−0.58264	KARPAS422, Lymphoma, DLBCL	−0.886750388	SUDHL4, Lymphoma, DLBCL	−0.981011953
11	KO52, Leukemia, AML	−0.57416	TE8, Esophageal Cancer	−0.837851667	SKPNDW, Bone Cancer	−0.979992815
12	SUDHL4, Lymphoma, DLBCL	−0.5526	GIMEN, Neuroblastoma	−0.823007681	TGW, Neuroblastoma	−0.940803109
13	TM87, Rhabdoid	−0.54929	AU565, Breast Cancer	−0.815466703	L1236, Lymphoma, B-cell, Hodgkins	−0.933248593
14	SLR23, Kidney Cancer	−0.51258	SNU349, Kidney Cancer	−0.813774633	TM87, Rhabdoid	−0.913530271
15	JMURTK2, Rhabdoid	−0.49178	BIN67, Ovarian Cancer	−0.806004209	IPC298, Skin Cancer	−0.911426595

## Data Availability

All relevant data are within the paper and its Supporting Information. Source code of the analysis can be found here: https://github.com/genesolution/PRC2_data (uploaded on 25 March 2021).

## Supplementary Materials

The following are available online at https://www.mdpi.com/article/10.3390/cancers13133155/s1, Supplementary File S1: CRISPR (AVANA) Public 20Q3 PRC2 cell lines info, Supplementary File S2: cBioPortal oncoprint representation of PRC2 alterations identified in different cancers, Supplementary File S3: Survival prognosis data for gastric cancer and thymoma, Supplementary File S4: PRC2 significant *p*-value_no_silent EZH2, SUZ12, EED.
