# Supplementary material for: Clinical Correlations of Polycomb Repressive Complex 2 in Different Tumor Types
Source: Cancers (Basel). 2021 Jun 24;13(13):3155. doi: 10.3390/cancers13133155 (PMC8267669; doi:10.3390/cancers13133155)
Supplement: Supplementary file 1 [file cancers-13-03155-s001.zip › Supplementary File S2. cBioPortal oncoprint representation of PRC2 alterations identified in different cancers.pdf]

cancers

Supplementary Materials: Clinical Correlations of Polycomb Repressive Complex 2 in Different Tumor Types

Maksim Erokhin, Olga Chetverina, Balázs Győrffy, Victor V. Tatarskiy, Vladic Mogila, Alexander A. Shtil, Igor B. Roninson, Jerome Moreaux, Pavel Georgiev, Giacomo Cavalli and Darya Chetverina

Supplementary File S2. cBioPortal oncoprint representation of PRC2 alterations identified in different cancers.

EZH2/SUZ12/EED Alterations in clinical samples

Data from cBioPortal database https://www.cbioportal.org/

Designations

Genetic Alteration

Inframe Mutation (unknown significance) Missense Mutation (putative driver) Missense Mutation (unknown significance) Truncating Mutation (putative driver) Amplification Deep Deletion No alterations Not profiled

# Samples per Patient 0 6

Profiled for copy number alterations Yes No

Profiled for mutations Yes No

Solid cancer

Ovary

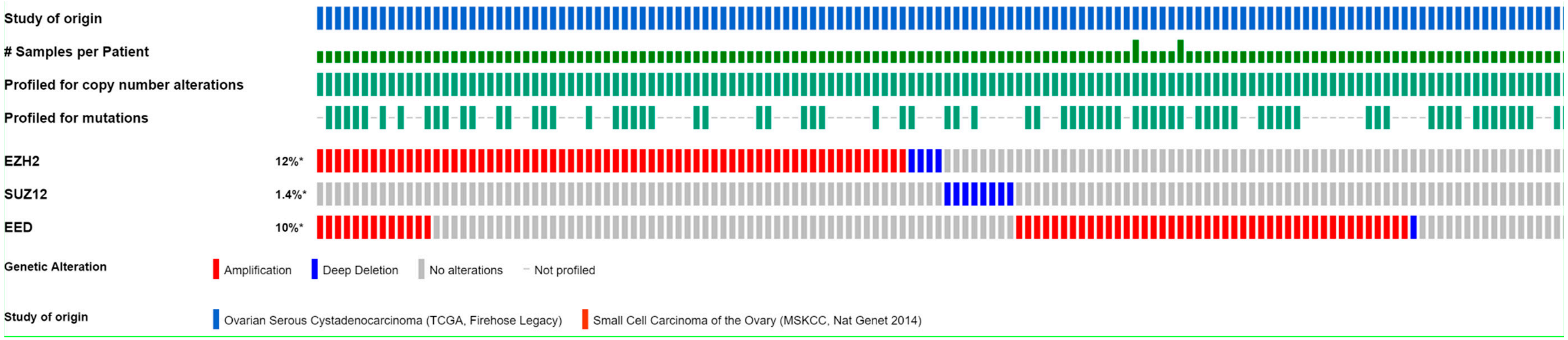

Skin

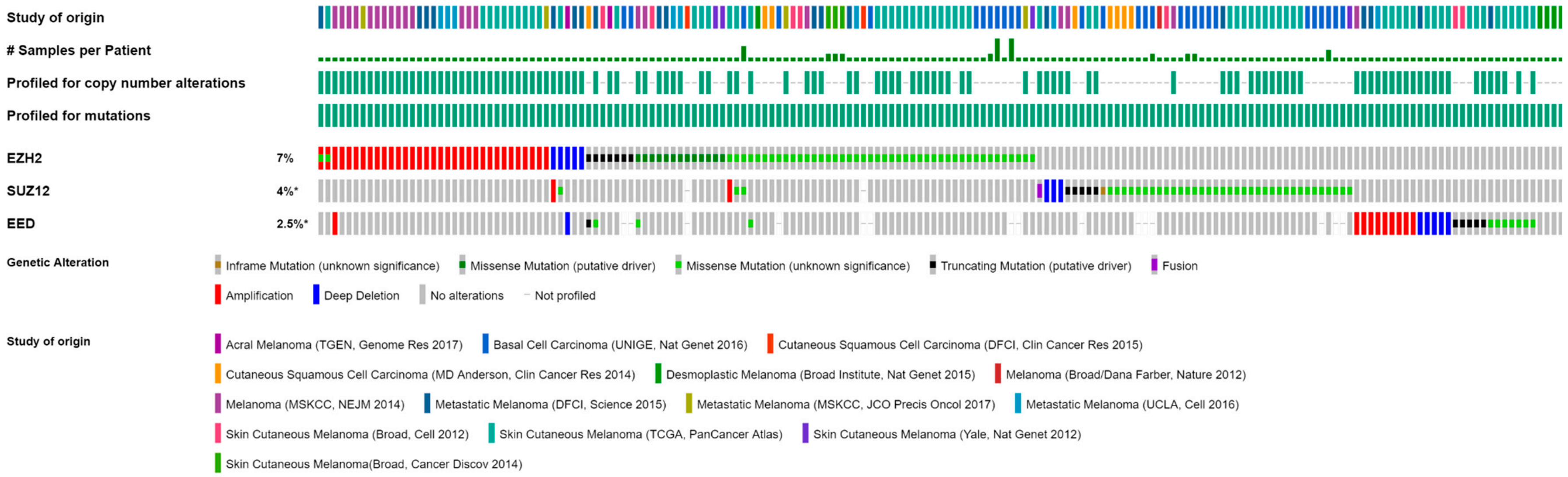

Soft Tissue

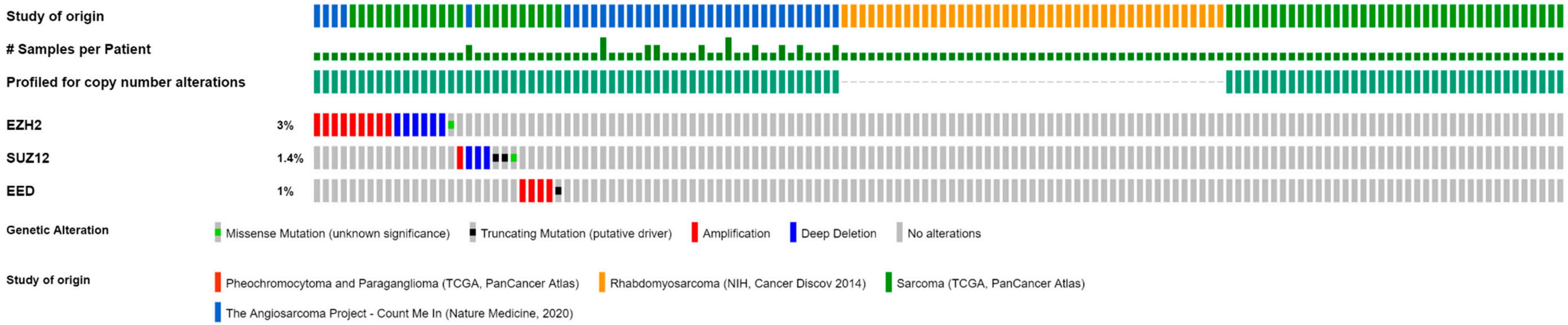

Prostate

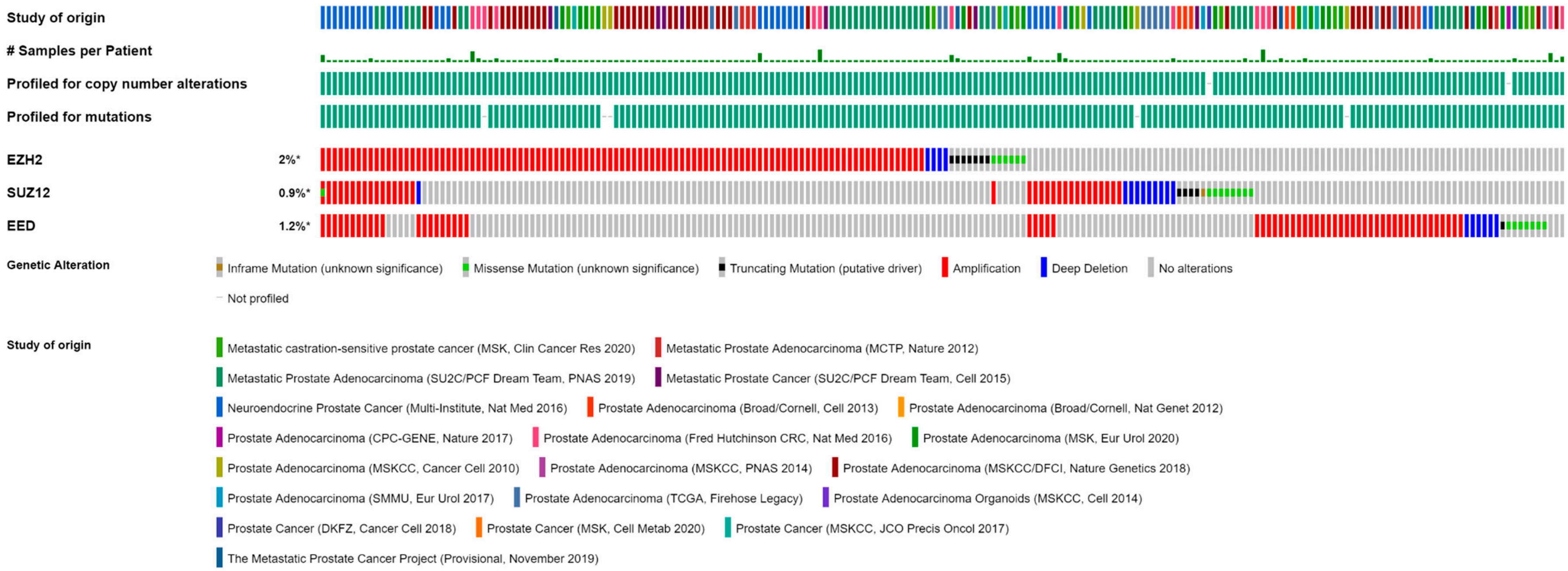
Study of origin
Samples per Patient
Profiled for copy number alterations
Profiled for mutations
EZH2 2%*
SUZ12 0.9%*
EED 1.2%*
Genetic Alteration
Inframe Mutation (unknown significance)
Missense Mutation (unknown significance)
Truncating Mutation (putative driver)
Amplification
Deep Deletion
No alterations
Not profiled
Study of origin
Metastatic castration-sensitive prostate cancer (MSK, Clin Cancer Res 2020)
Metastatic Prostate Adenocarcinoma (MCTP, Nature 2012)
Metastatic Prostate Adenocarcinoma (SU2C/PCF Dream Team, PNAS 2019)
Metastatic Prostate Cancer (SU2C/PCF Dream Team, Cell 2015)
Neuroendocrine Prostate Cancer (Multi-Institute, Nat Med 2016)
Prostate Adenocarcinoma (Broad/Cornell, Cell 2013)
Prostate Adenocarcinoma (Broad/Cornell, Nat Genet 2012)
Prostate Adenocarcinoma (CPC-GENE, Nature 2017)
Prostate Adenocarcinoma (Fred Hutchinson CRC, Nat Med 2016)
Prostate Adenocarcinoma (MSK, Eur Urol 2020)
Prostate Adenocarcinoma (MSKCC, Cancer Cell 2010)
Prostate Adenocarcinoma (MSKCC, PNAS 2014)
Prostate Adenocarcinoma (MSKCC/DFCI, Nature Genetics 2018)
Prostate Adenocarcinoma (SMMU, Eur Urol 2017)
Prostate Adenocarcinoma (TCGA, Firehose Legacy)
Prostate Adenocarcinoma Organoids (MSKCC, Cell 2014)
Prostate Cancer (DKFZ, Cancer Cell 2018)
Prostate Cancer (MSK, Cell Metab 2020)
Prostate Cancer (MSKCC, JCO Precis Oncol 2017)
The Metastatic Prostate Cancer Project (Provisional, November 2019)

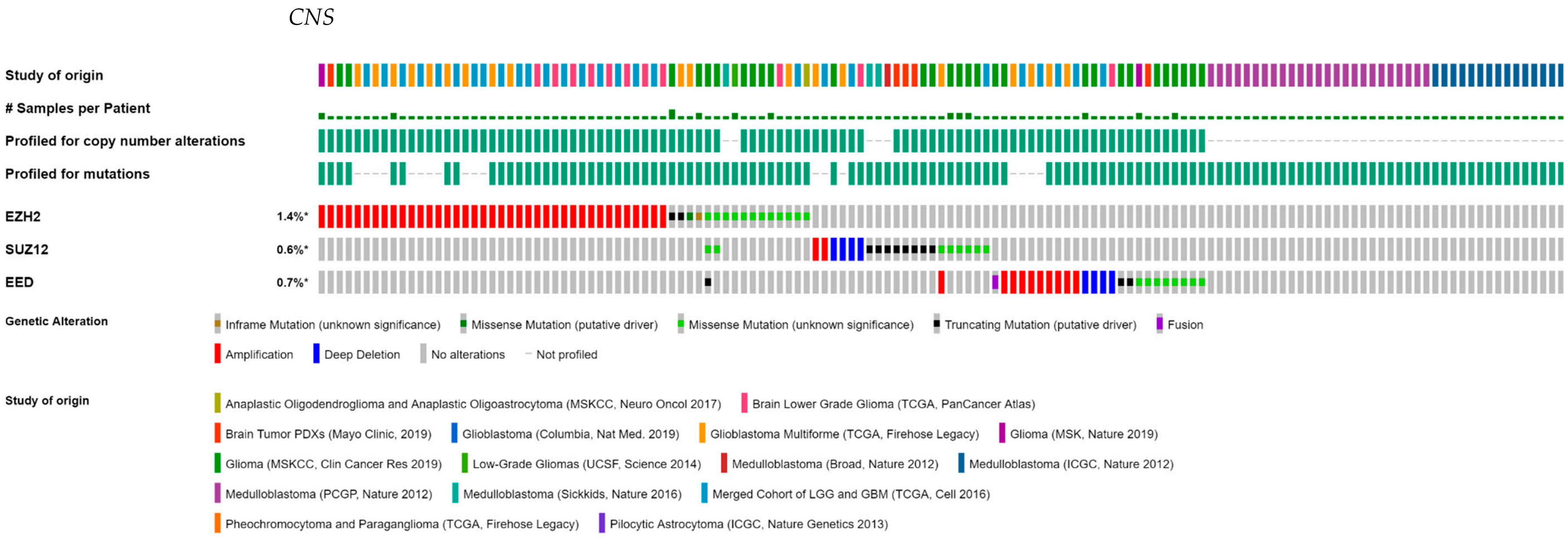
CNS
Study of origin
Samples per Patient
Profiled for copy number alterations
Profiled for mutations
EZH2 1.4%*
SUZ12 0.6%*
EED 0.7%*
Genetic Alteration
Inframe Mutation (unknown significance)
Missense Mutation (putative driver)
Missense Mutation (unknown significance)
Truncating Mutation (putative driver)
Fusion
Amplification
Deep Deletion
No alterations
Not profiled
Study of origin
Anaplastic Oligodendroglioma and Anaplastic Oligoastrocytoma (MSKCC, Neuro Oncol 2017)
Brain Lower Grade Glioma (TCGA, PanCancer Atlas)
Brain Tumor PDXs (Mayo Clinic, 2019)
Glioblastoma (Columbia, Nat Med. 2019)
Glioblastoma Multiforme (TCGA, Firehose Legacy)
Glioma (MSK, Nature 2019)
Glioma (MSKCC, Clin Cancer Res 2019)
Low-Grade Gliomas (UCSF, Science 2014)
Medulloblastoma (Broad, Nature 2012)
Medulloblastoma (ICGC, Nature 2012)
Medulloblastoma (PCGP, Nature 2012)
Medulloblastoma (Sickkids, Nature 2016)
Merged Cohort of LGG and GBM (TCGA, Cell 2016)
Pheochromocytoma and Paraganglioma (TCGA, Firehose Legacy)
Pilocytic Astrocytoma (ICGC, Nature Genetics 2013)

Breast

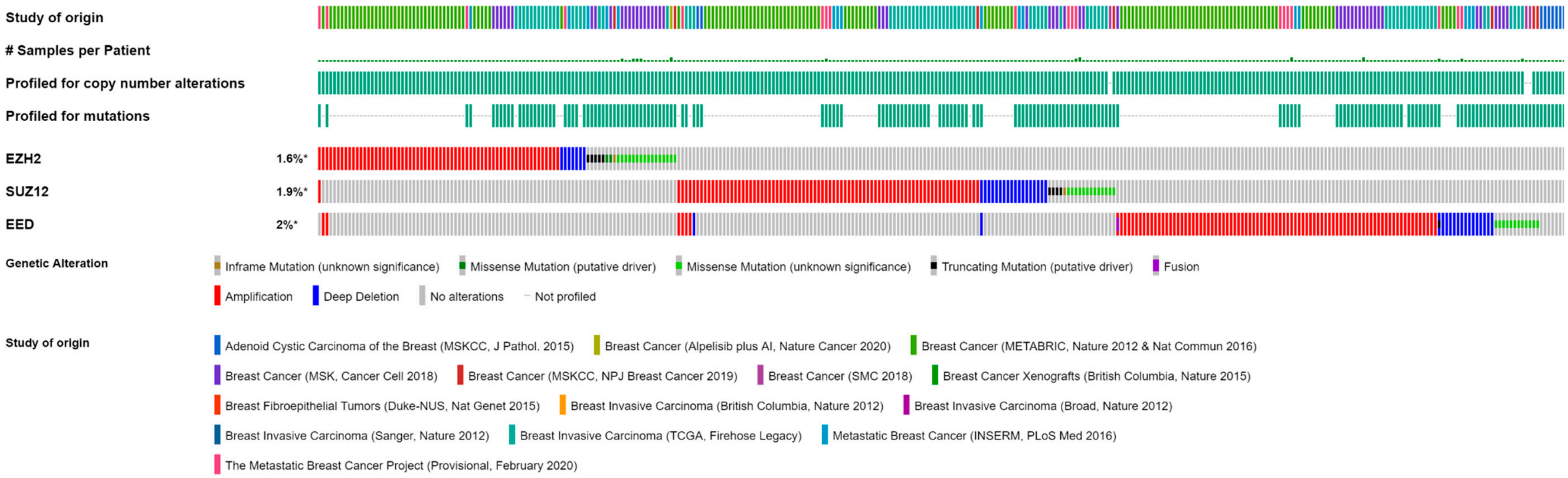
Study of origin
Samples per Patient
Profiled for copy number alterations
Profiled for mutations
EZH2
1.6%*
SUZ12
1.9%*
EED
2%*
Genetic Alteration
Inframe Mutation (unknown significance)
Missense Mutation (putative driver)
Missense Mutation (unknown significance)
Truncating Mutation (putative driver)
Fusion
Amplification
Deep Deletion
No alterations
Not profiled
Study of origin
Adenoid Cystic Carcinoma of the Breast (MSKCC, J Pathol. 2015)
Breast Cancer (Alpelisib plus AI, Nature Cancer 2020)
Breast Cancer (METABRIC, Nature 2012 & Nat Commun 2016)
Breast Cancer (MSK, Cancer Cell 2018)
Breast Cancer (MSKCC, NPJ Breast Cancer 2019)
Breast Cancer (SMC 2018)
Breast Cancer Xenografts (British Columbia, Nature 2015)
Breast Fibroepithelial Tumors (Duke-NUS, Nat Genet 2015)
Breast Invasive Carcinoma (British Columbia, Nature 2012)
Breast Invasive Carcinoma (Broad, Nature 2012)
Breast Invasive Carcinoma (Sanger, Nature 2012)
Breast Invasive Carcinoma (TCGA, Firehose Legacy)
Metastatic Breast Cancer (INSERM, PLoS Med 2016)
The Metastatic Breast Cancer Project (Provisional, February 2020)

Kidney

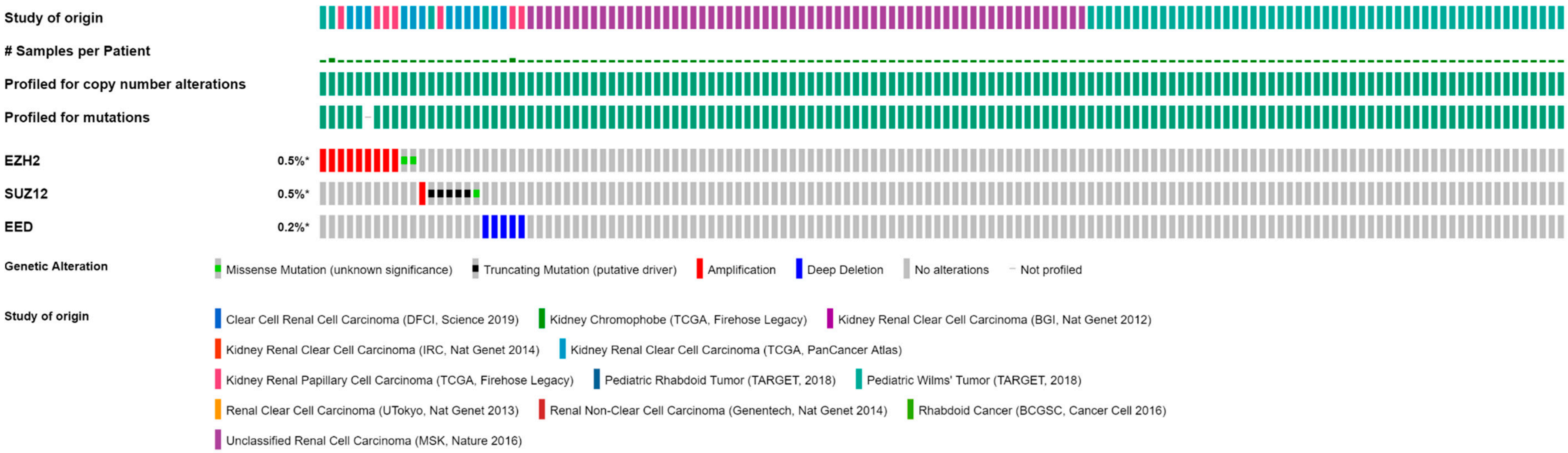
Study of origin
Samples per Patient
Profiled for copy number alterations
Profiled for mutations
EZH2
0.5%*
SUZ12
0.5%*
EED
0.2%*
Genetic Alteration
Missense Mutation (unknown significance)
Truncating Mutation (putative driver)
Amplification
Deep Deletion
No alterations
Not profiled
Study of origin
Clear Cell Renal Cell Carcinoma (DFCI, Science 2019)
Kidney Chromophobe (TCGA, Firehose Legacy)
Kidney Renal Clear Cell Carcinoma (BGI, Nat Genet 2012)
Kidney Renal Clear Cell Carcinoma (IRC, Nat Genet 2014)
Kidney Renal Clear Cell Carcinoma (TCGA, PanCancer Atlas)
Kidney Renal Papillary Cell Carcinoma (TCGA, Firehose Legacy)
Pediatric Rhabdoid Tumor (TARGET, 2018)
Pediatric Wilms' Tumor (TARGET, 2018)
Renal Clear Cell Carcinoma (UTokyo, Nat Genet 2013)
Renal Non-Clear Cell Carcinoma (Genentech, Nat Genet 2014)
Rhabdoid Cancer (BCGSC, Cancer Cell 2016)
Unclassified Renal Cell Carcinoma (MSK, Nature 2016)

Lung

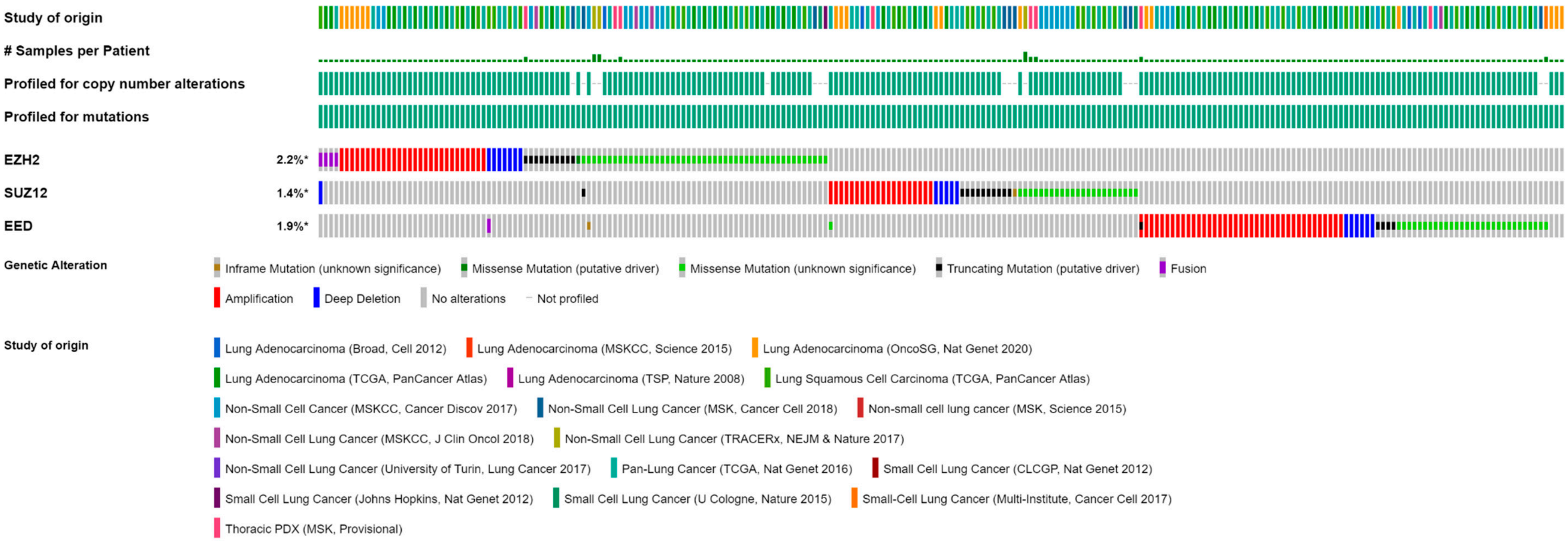
Study of origin
Samples per Patient
Profiled for copy number alterations
Profiled for mutations
EZH2
2.2%*
SUZ12
1.4%*
EED
1.9%*
Genetic Alteration
Inframe Mutation (unknown significance)
Missense Mutation (putative driver)
Missense Mutation (unknown significance)
Truncating Mutation (putative driver)
Fusion
Amplification
Deep Deletion
No alterations
Not profiled
Study of origin
Lung Adenocarcinoma (Broad, Cell 2012)
Lung Adenocarcinoma (MSKCC, Science 2015)
Lung Adenocarcinoma (OncoSG, Nat Genet 2020)
Lung Adenocarcinoma (TCGA, PanCancer Atlas)
Lung Adenocarcinoma (TSP, Nature 2008)
Lung Squamous Cell Carcinoma (TCGA, PanCancer Atlas)
Non-Small Cell Cancer (MSKCC, Cancer Discov 2017)
Non-Small Cell Lung Cancer (MSK, Cancer Cell 2018)
Non-small cell lung cancer (MSK, Science 2015)
Non-Small Cell Lung Cancer (MSKCC, J Clin Oncol 2018)
Non-Small Cell Lung Cancer (TRACERx, NEJM & Nature 2017)
Non-Small Cell Lung Cancer (University of Turin, Lung Cancer 2017)
Pan-Lung Cancer (TCGA, Nat Genet 2016)
Small Cell Lung Cancer (CLCGP, Nat Genet 2012)
Small Cell Lung Cancer (Johns Hopkins, Nat Genet 2012)
Small Cell Lung Cancer (U Cologne, Nature 2015)
Small-Cell Lung Cancer (Multi-Institute, Cancer Cell 2017)
Thoracic PDX (MSK, Provisional)

Bladder/Urinary tract

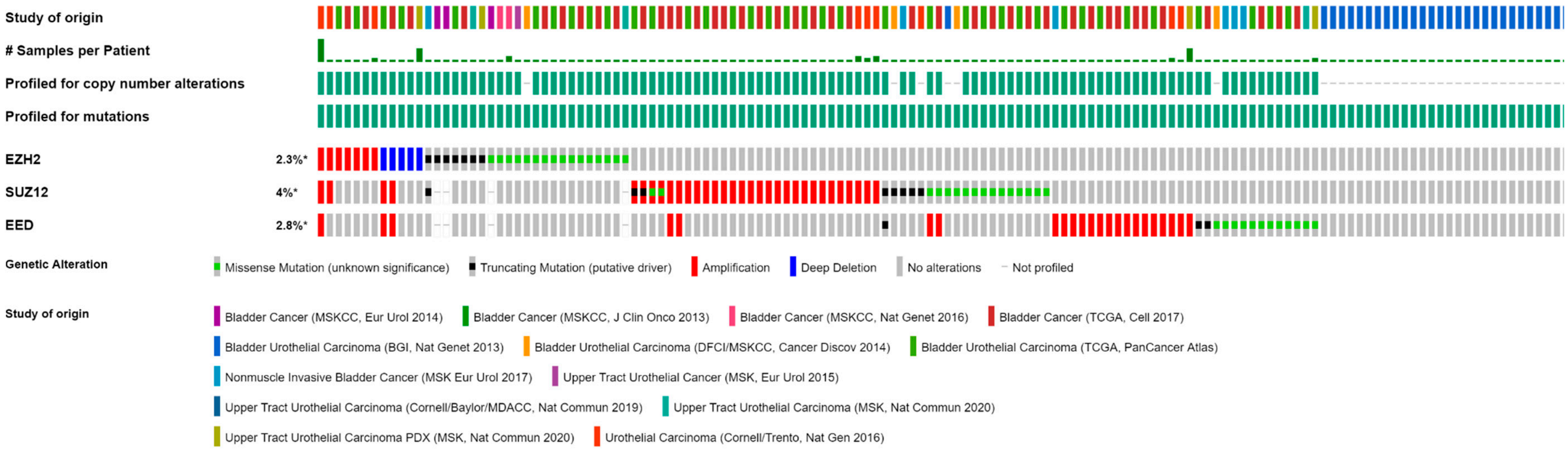

Esophagus/Stomach

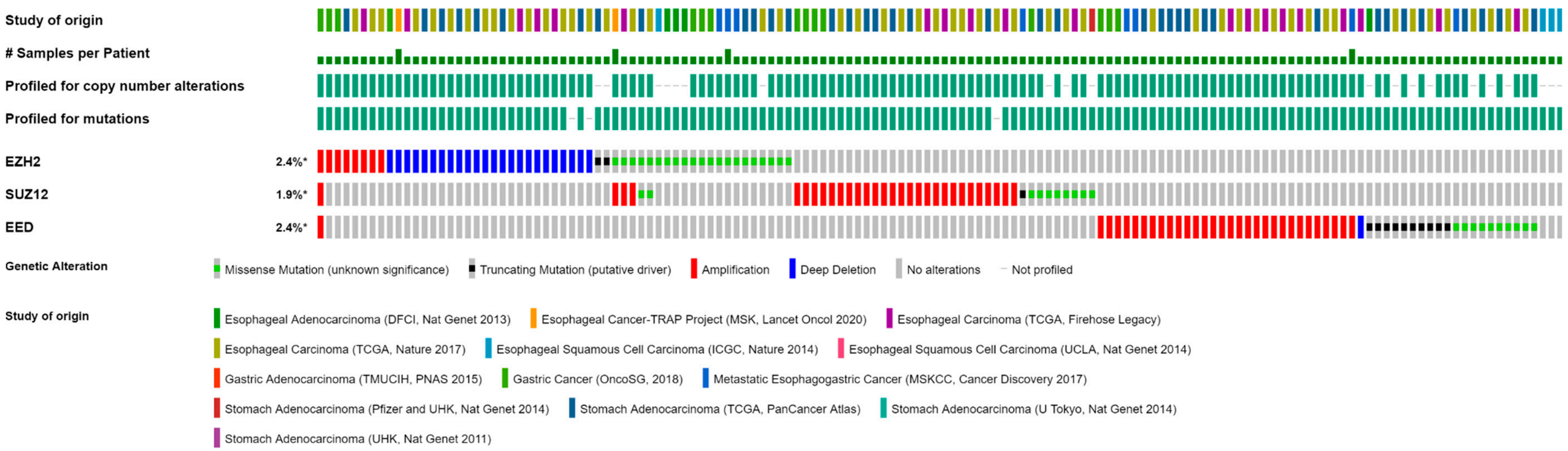

Uterus

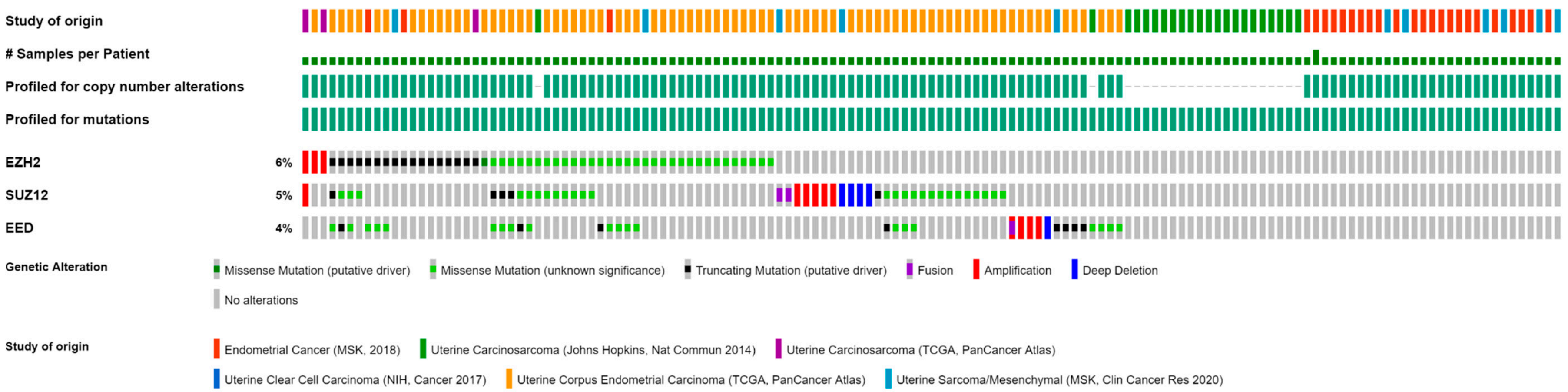

Liver

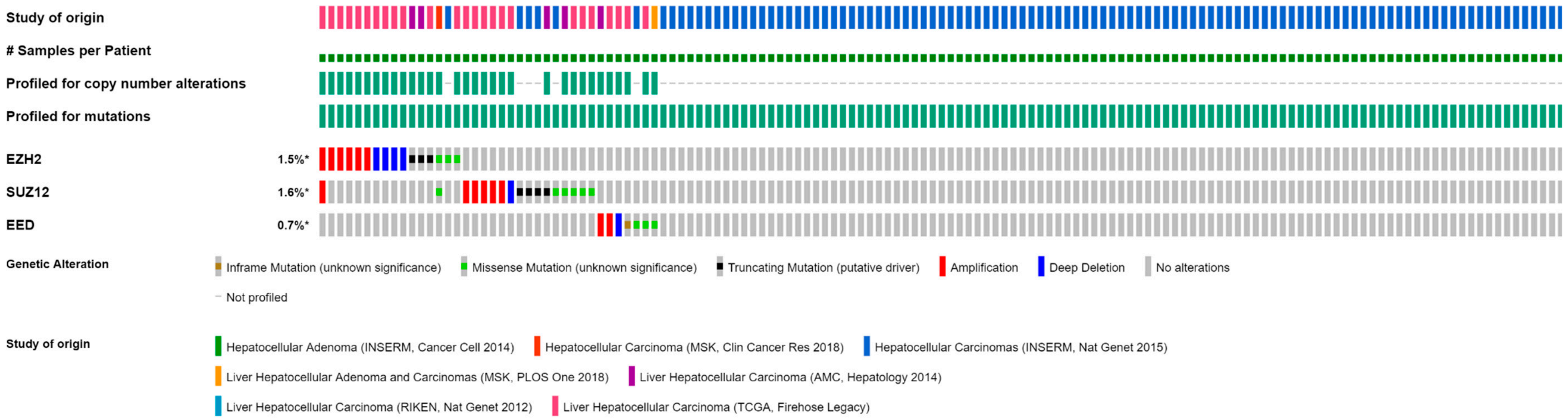

Head and neck

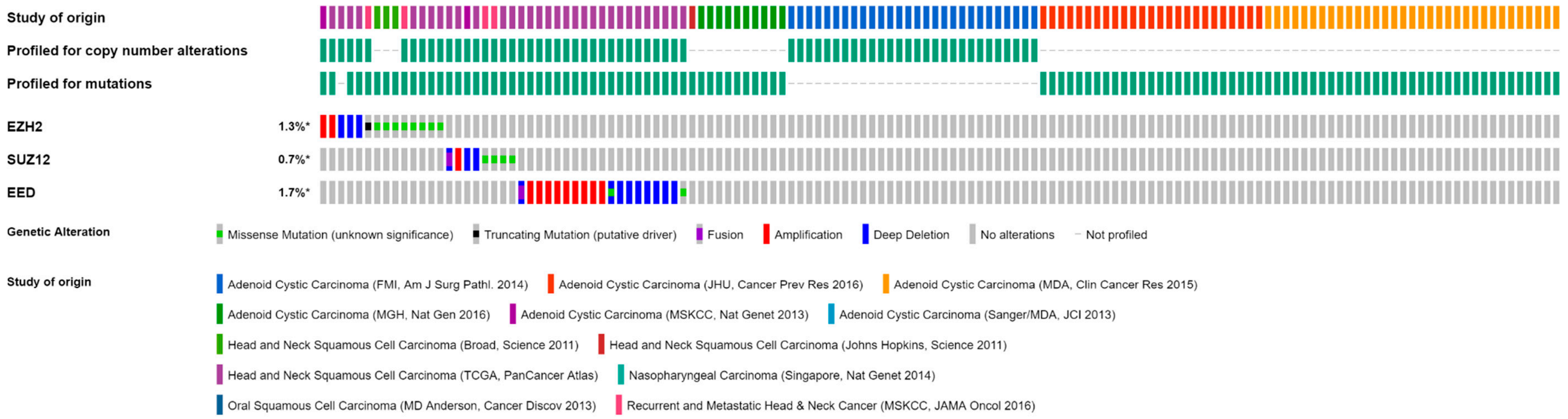

Adrenal gland

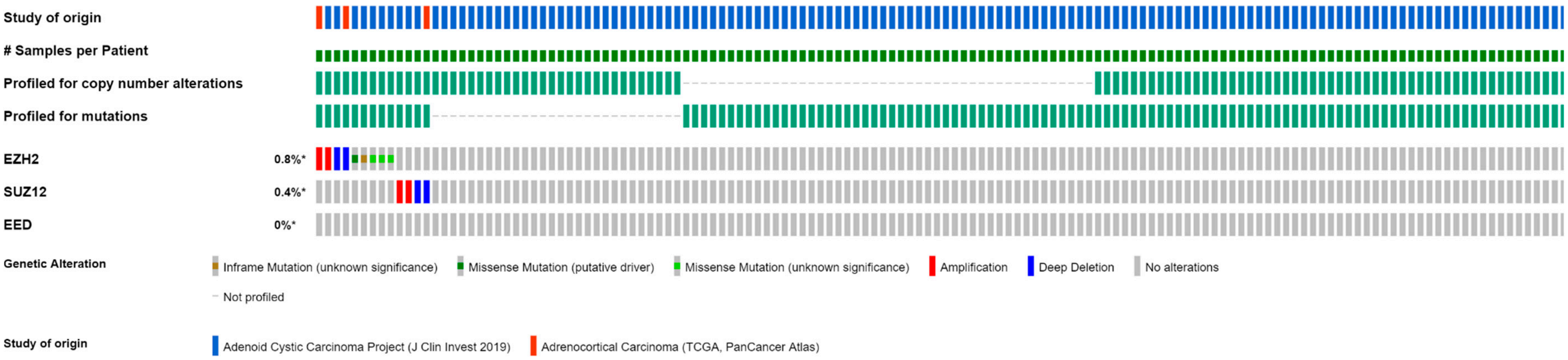

Bowel

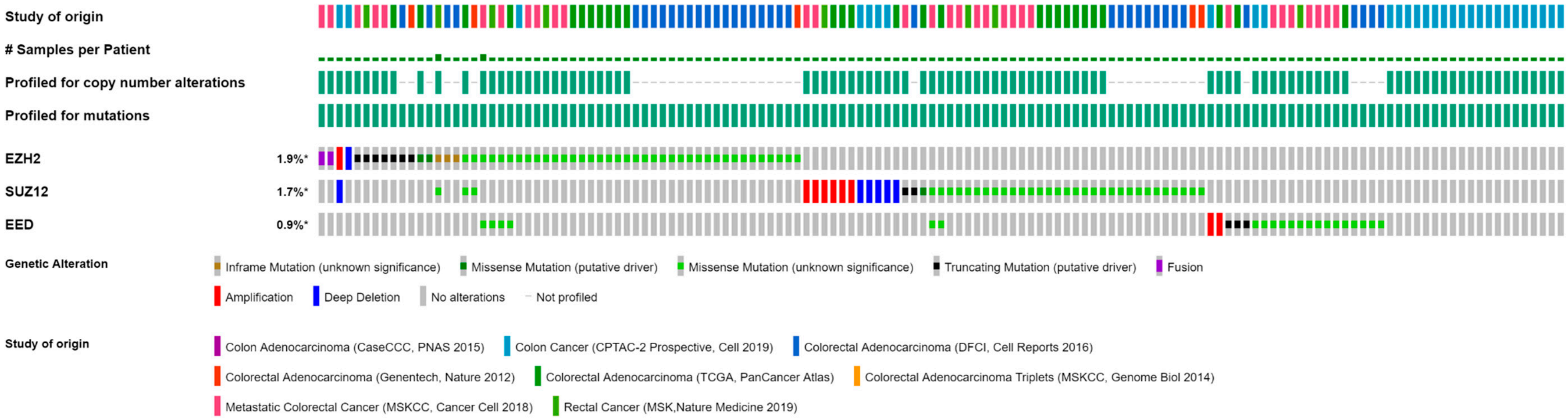

Blood

Lymphoid

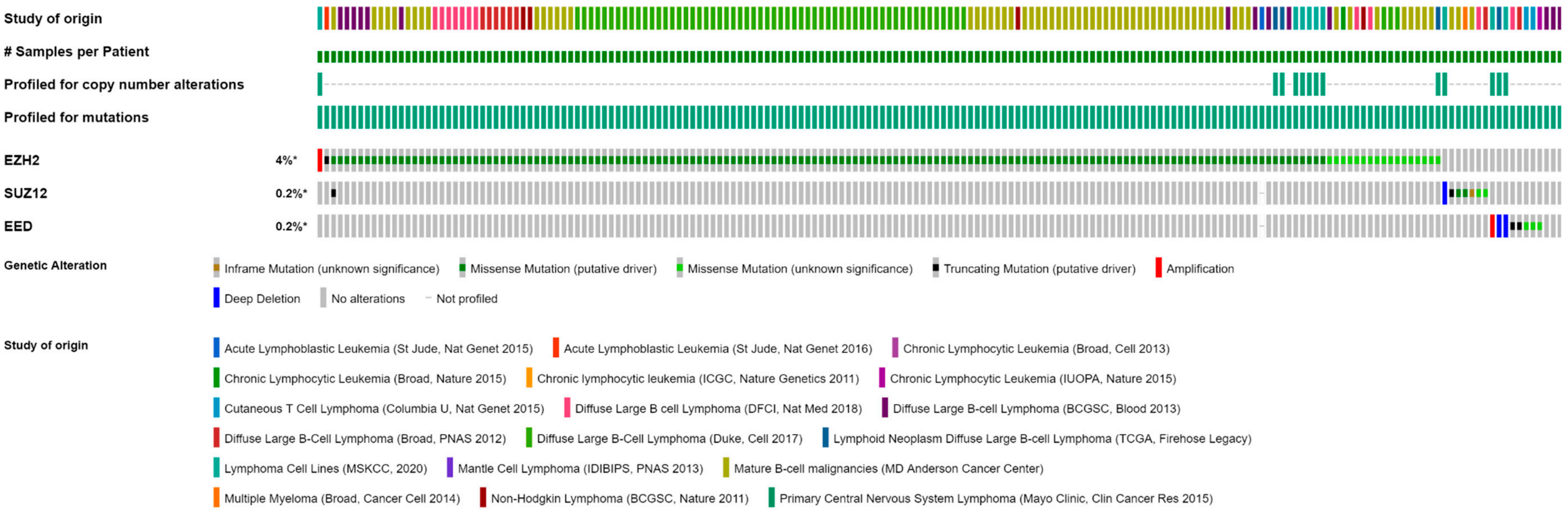

DLBCL

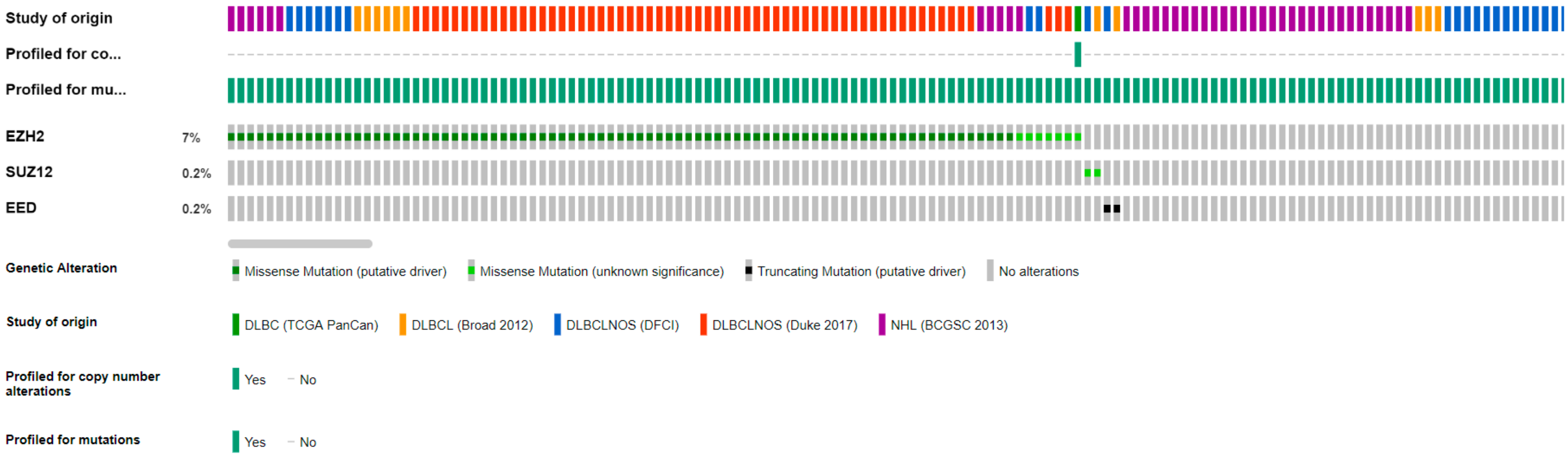
Study of origin
Profiled for co...
Profiled for mu...
EZH2 7%
SUZ12 0.2%
EED 0.2%
Genetic Alteration
Missense Mutation (putative driver)
Missense Mutation (unknown significance)
Truncating Mutation (putative driver)
No alterations
Study of origin
DLBC (TCGA PanCan)
DLBCL (Broad 2012)
DLBCLNOS (DFCI)
DLBCLNOS (Duke 2017)
NHL (BCGSC 2013)
Profiled for copy number alterations
Yes
No
Profiled for mutations
Yes
No

Myeloid

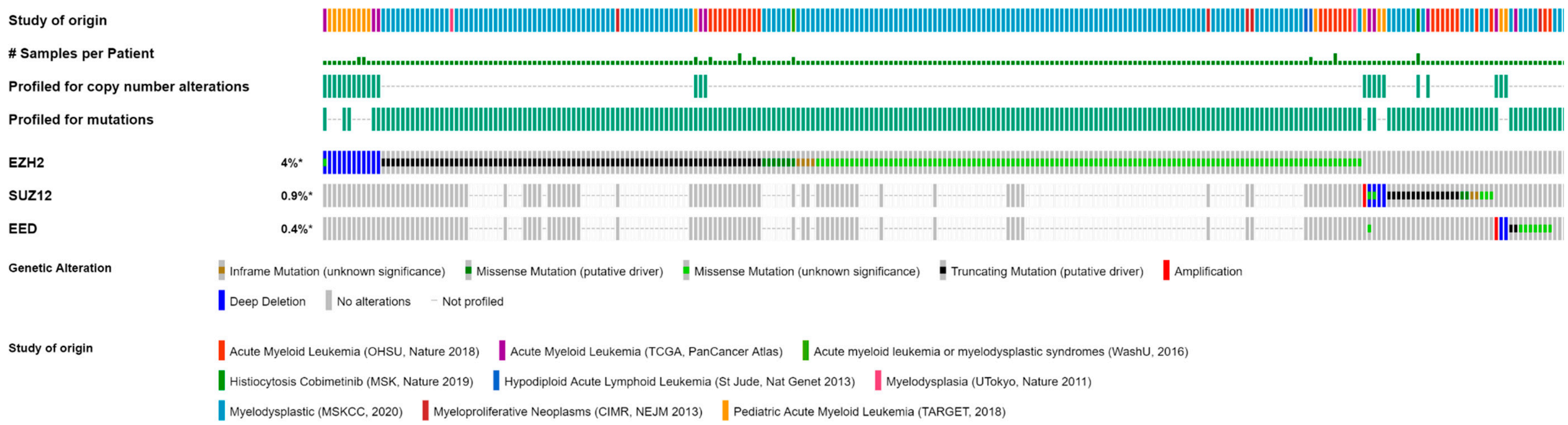
Study of origin
Samples per Patient
Profiled for copy number alterations
Profiled for mutations
EZH2 4%*
SUZ12 0.9%*
EED 0.4%*
Genetic Alteration
Inframe Mutation (unknown significance)
Missense Mutation (putative driver)
Missense Mutation (unknown significance)
Truncating Mutation (putative driver)
Amplification
Deep Deletion
No alterations
Not profiled
Study of origin
Acute Myeloid Leukemia (OHSU, Nature 2018)
Acute Myeloid Leukemia (TCGA, PanCancer Atlas)
Acute myeloid leukemia or myelodysplastic syndromes (WashU, 2016)
Histiocytosis Cobimetinib (MSK, Nature 2019)
Hypodiploid Acute Lymphoid Leukemia (St Jude, Nat Genet 2013)
Myelodysplasia (UTokyo, Nature 2011)
Myelodysplastic (MSKCC, 2020)
Myeloproliferative Neoplasms (CIMR, NEJM 2013)
Pediatric Acute Myeloid Leukemia (TARGET, 2018)
