# Supplementary material for: Clinical Correlations of Polycomb Repressive Complex 2 in Different Tumor Types
Source: Cancers (Basel). 2021 Jun 24;13(13):3155. doi: 10.3390/cancers13133155 (PMC8267669; doi:10.3390/cancers13133155)
Supplement: Supplementary file 1 [file cancers-13-03155-s001.zip › Supplementary File S3. Survival prognosis data for gastric cancer and thymoma.pdf]

cancers

MDPI

Supplementary Materials: Clinical Correlations of Polycomb Repressive Complex 2 in Different Tumor Types

Maksim Erokhin, Olga Chetverina, Balázs Győrffy, Victor V. Tatarskiy, Vladic Mogila, Alexander A. Shtil, Igor B. Roninson, Jerome Moreaux, Pavel Georgiev, Giacomo Cavalli and Darya Chetverina

Supplementary File S3. Survival prognosis data for gastric cancer and thymoma for proof (Erokhin et al).

This file contains the correlation data between the overall survival and the level of *EZH2*, *SUZ12* or *EED* transcription for gastric cancer and thymoma.

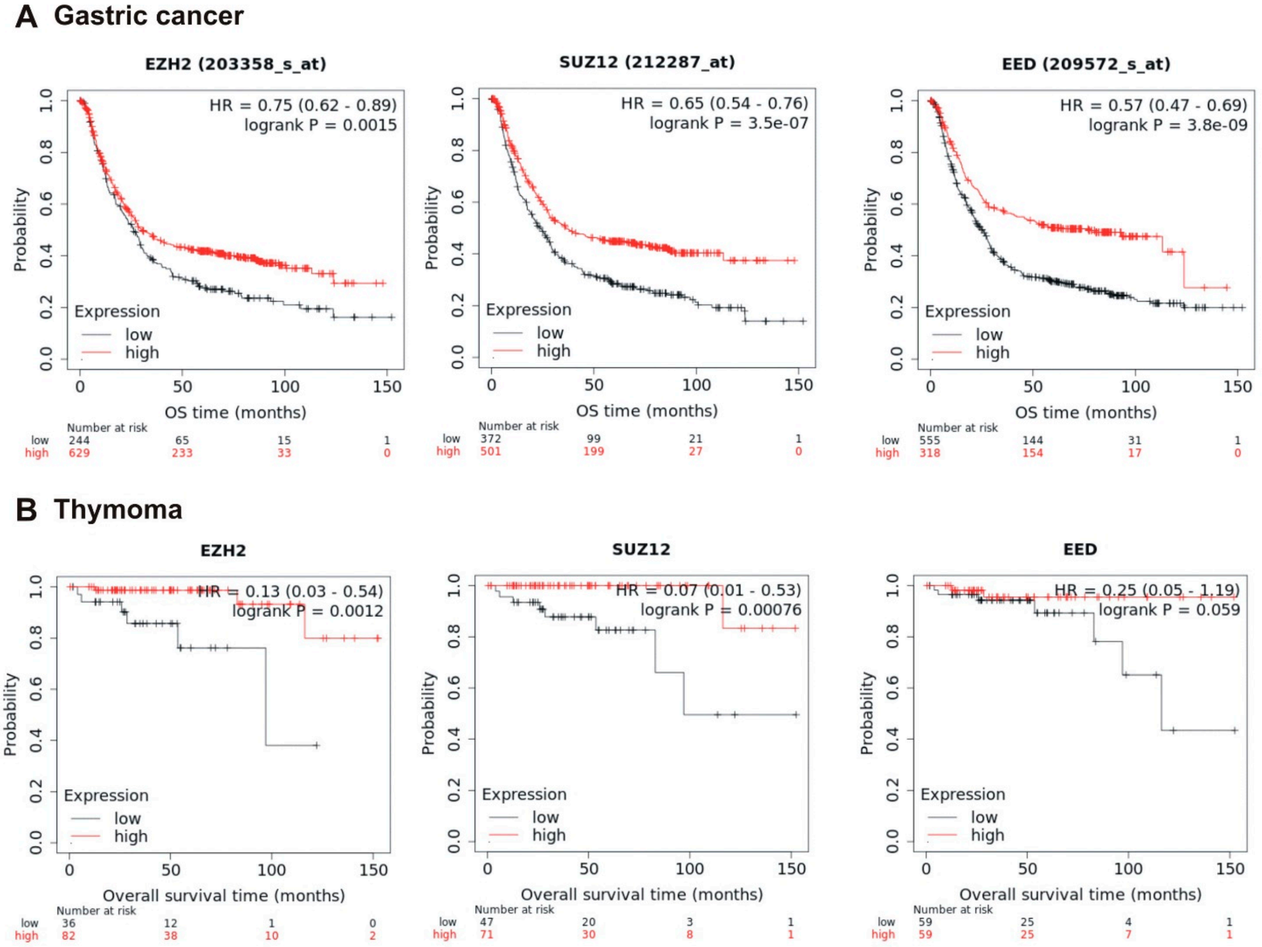

Figure S1. The overexpression of either of the PRC2 genes correlated with a longer patient survival in gastric cancer (A) and thymoma (B). The analysis was performed using KMplot resource. The cohort with low level of gene expression is colored in black, cohort with high expression is colored in red.
